# Effects of concurrent training on exercise capacity and quality of life in older adult patients with COPD: a Bayesian pairwise and dose–response meta-analysis

**DOI:** 10.3389/fmed.2026.1760792

**Published:** 2026-04-09

**Authors:** Shengnian Li, Yikun Yin, Jingjun Guan, Liang Yu

**Affiliations:** 1 School of Strength and Conditioning Training, Beijing Sport University, Beijing, China; 2 Sport Science School, Beijing Sport University, Beijing, China; 3 Academic Affairs Office, Beijing Sport University, Beijing, China

**Keywords:** concurrent training, COPD, dose–response meta-analysis, exercise capacity, quality of life

## Abstract

**Objective:**

This systematic review and meta-analysis aimed to assess the effect of concurrent training (CT) on exercise capacity and quality of life in patients with chronic obstructive pulmonary disease (COPD), and to identify the optimal CT dose to enhance 6-min walk distance (6MWD).

**Methods:**

Relevant randomized controlled trials (RCTs) examining the effects of CT on exercise capacity and quality of life in patients with COPD were identified through a comprehensive search of PubMed, Embase, Web of Science, Cochrane Library, Scopus, and SPORTDiscus. A multilevel Bayesian random-effects model was used to conduct both pairwise and dose–response meta-analyses.

**Results:**

A total of 1,037 COPD patients were included in the 20 studies. Based on pairwise comparisons, CT was found to significantly improve 6MWD (MD: 44.08; 95% CrI: 33.35–54.72; SD: 20.85; 95% CrI: 13.29–32.26), VO_2max_ (MD: 1.02; 95% CrI: 0.04–2.00; SD: 0.91; 95% CrI: 0.23–2.12), LP 1RM (MD: 30.53; 95% CrI: 3.38–57.71; SD: 2.52; 95% CrI: 0.04–15.19), CP 1RM (MD: 12.20; 95% CrI: 2.77–21.59; SD: 2.44; 95% CrI: 0.05–10.71), and SGRQ score (MD: −8.65; 95% CrI: −10.79 to −6.51; SD: 5.04; 95% CrI: 2.52–8.99). However, no significant improvement was observed in FVC, FEV_1_, and FEV_1_/FVC. Additionally, a nonlinear dose–response relationship was observed between CT and 6MWD, with the optimal dose identified as 1,220 MET-min/week (MD = 24.83; 95% CrI: 14.96–34.70).

**Conclusions:**

CT was found to significantly improve exercise capacity and quality of life in COPD patients, while showing limited effects on pulmonary function indicators. Moreover, a nonlinear dose–response relationship was identified between CT and 6MWD, with the most pronounced effects observed at a weekly dose of 1,220 MET-min.

**Systematic review registration:**

https://www.crd.york.ac.uk/prospero/, CRD42025630487.

## 1 Introduction

Chronic Obstructive Pulmonary Disease (COPD) is a prevalent chronic respiratory condition characterized by persistent airflow limitation caused by abnormalities in the airways and alveolar structure (1). According to the World Health Organization (WHO), COPD was the fourth leading cause of death worldwide in 2021, contributing to approximately 3.5 million deaths, or nearly 5% of all global fatalities. By 2060, COPD is projected to cause approximately 5.4 million deaths (2), posing a major global public health challenge (3). In the context of population aging, successful aging is defined as the ability to maintain a good quality of life in older age (4). As a primary health concern among older adults, COPD is closely associated with the health challenges related to population aging. Statistics indicate that the prevalence of COPD reaches up to 16% among individuals over the age of 65 years (5), highlighting the urgent need for targeted interventions.

Pulmonary rehabilitation (PR) is a systematic and evidence-based comprehensive intervention strategy, particularly suitable for middle-aged and older patients with COPD (6). PR includes exercise training (7), health education (8), nutritional guidance (9), and psychological support (10). Among these components, exercise training is considered one of the most cost-effective non-pharmacological interventions (11), and common forms include aerobic training (12), interval training (13), resistance training (14), and inspiratory muscle training (15). Previous studies have shown that aerobic training mainly targets the cardiopulmonary system and can significantly improve cardiovascular adaptability, oxidative metabolism, and aerobic capacity. However, its effects on skeletal muscle atrophy and dysfunction are limited, and these factors are key contributors to exercise intolerance and reduced quality of life in patients with COPD (16). Resistance training mainly targets the neuromuscular system and can increase muscle strength and muscle mass by activating muscle protein synthesis pathways, thereby helping to prevent skeletal muscle atrophy. These two training modalities show complementary advantages in terms of physiological adaptations. However, studies investigating the effects of concurrent training (CT) on exercise capacity and quality of life in patients with COPD remain limited, and evidence regarding the dose-response relationship is still lacking. Therefore, identifying the optimal training dose of CT has become an important issue that needs to be addressed in current clinical practice.

At present, most meta-analyses related to COPD mainly focus on the effects of aerobic training or resistance training alone on exercise capacity and quality of life, while relatively few studies have examined these outcomes from the perspective of concurrent training. The effectiveness of CT interventions is influenced by factors such as training intensity, training volume, and intervention duration, and different training parameters may induce different physiological adaptations. Therefore, clarifying the dose–response relationship of different training parameters in patients with COPD has important clinical significance. In addition, whether CT can significantly improve exercise capacity and quality of life in patients with COPD remains controversial (17, 18), and existing meta-analyses lack in-depth exploration of potential moderating variables such as age, sex, and BMI (19–21). Based on this, the present study conducted Bayesian pairwise and dose-response meta-analyses to compare the effects of different intervention doses of CT on exercise capacity and quality of life in patients with COPD. In addition, potential moderating factors including age, sex, and BMI were further examined to provide evidence-based support for the development of scientific and individualized exercise intervention strategies.

## 2 Methods

This study was conducted in accordance with the Preferred Reporting Items for Systematic Reviews and Meta-Analyses (PRISMA) guidelines (22) and the Cochrane Handbook for Systematic Reviews of Interventions (23). The study protocol was registered in advance on PROSPERO (CRD42025630487). Ethical approval was not required for this meta-analysis, as all data were derived from previously published studies.

### 2.1 Search strategy

A systematic search was conducted across PubMed, Embase, Web of Science, Cochrane Library, Scopus, and SPORTDiscus to identify all relevant literature published from the database inception to February 20, 2025. The search strategy involved both Medical Subject Headings and free-text keywords. The main search terms included “Pulmonary Disease, Chronic Obstructive,” “Chronic Obstructive Pulmonary Diseases,” “COPD,” “Exercise,” “Physical Activity,” and “Exercise Training.” The search was restricted to English-language publications, with no regional limitations. Following the final selection of eligible studies, reference lists were manually screened to ensure no relevant literature was overlooked. The search strategy is presented in Supplementary Appendix 1.

### 2.2 Eligibility criteria

The inclusion criteria were as follows:

#### 2.2.1 Participants

Older adult patients aged 55 years and above with a diagnosis of COPD (24).

#### 2.2.2 Interventions

The intervention group participated in concurrent aerobic and resistance training for at least 8 weeks (25), as recommended by the American Thoracic Society (ATS), which suggests that a minimum of 8 weeks is required to achieve and maintain improvements in exercise capacity and quality of life among patients with COPD (26).

#### 2.2.3 Comparisons

The control group received non-exercise interventions, including usual care, wait-list control, or daily activities.

#### 2.2.4 Outcomes

Studies were required to report at least one of the following outcomes.

- Cardiopulmonary fitness: 6-min walk distance (6MWD), maximal oxygen uptake (VO_2max_), endurance shuttle walk test (ESWT);
- Pulmonary function: forced vital capacity (FVC), forced expiratory volume in 1 s (FEV_1_), FEV_1_/FVC;
- Muscle strength: leg press 1RM (LP), chest press 1RM (CP), peak work rate (Wpeak);
- Quality of life: St. George's Respiratory Questionnaire (SGRQ).

#### 2.2.5 Study

Randomized Controlled Trials (RCTs).

The exclusion criteria were as follows:

- Qualitative studies, conference papers, systematic reviews or meta-analyses, study protocols, and gray literature;
- Animal studies;
- Duplicate publications or studies lacking extractable outcome data.

### 2.3 Study selection

The literature screening process was performed using EndNote 20 software (Clarivate Analytics, https://endnote.com). Two reviewers independently assessed the titles, abstracts, and full texts of all studies. For studies that could not be excluded based solely on title and abstract, the full texts were further reviewed. Any discrepancies were resolved by consulting a third reviewer with relevant expertise.

### 2.4 Data extraction

Data extraction was independently performed by two reviewers, and the results were cross-checked for consistency. Any discrepancies were resolved by consulting a third reviewer with relevant expertise. The extracted information included the first author, year of publication, participant characteristics (e.g., age, gender, sample size, BMI), intervention methods and components (type of intervention, exercise intensity, duration, frequency), and outcome measures (6MWD, VO_2max_, ESWT, FVC, FEV_1_, FEV_1_/FVC, LP 1RM, CP 1RM, Wpeak, SGRQ). If outcome data could not be obtained directly from the text, the corresponding author was contacted. If no response was received and relevant data were presented graphically, Web Plot Digitizer 4.1 (Ankit Rohatgi, https://apps.automeris.io) was utilized to extract the data.

### 2.5 Risk of bias and quality of evidence

Two reviewers independently evaluated the quality of the included studies, and any disagreements were resolved through a cross-checking process. When disagreements arose, a third reviewer with relevant expertise was consulted to reach consensus. The risk of bias was independently evaluated using the second version of the Cochrane Risk of Bias tool (RoB 2) (Cochrane Collaboration, https://methods.cochrane.org), which assesses five domains: the randomization process, deviations from intended interventions, missing outcome data, measurement of the outcome, and selection of the reported result. Each domain was assessed using predefined responses (“Yes,” “Probably Yes,” or “No”) to determine the overall risk of bias, which was categorized as low risk, some concerns, or high risk (27). The quality of evidence was graded using the Grading of Recommendations, Assessment, Development, and Evaluation (GRADE) system, which classifies evidence into four levels: high, moderate, low, and very low (28).

### 2.6 Statistical analysis

#### 2.6.1 Pairwise meta-analyses and publication bias

The mean difference (MD) and standard deviation (SD) of baseline changes for each outcome measure were primarily extracted. When these data were not provided, the standard error (SE) and interquartile range were used to estimate the corresponding SD (29). Bayesian meta-analyses were conducted using the “brms” package in R to examine the effects of CT on patients with COPD. This package offers flexible modeling options and facilitates intuitive interpretation through probabilistic inference (30). The Bayesian hierarchical models were constructed with study-level nesting of effect sizes, and posterior distributions were derived for all estimates (31). A weakly informative prior was specified for the intercept [μ ~ Normal (0,1)], the between-study heterogeneity parameter Tau was modeled with a Normal (0,1) prior (32), and the sigma parameter was modeled with a Half-Cauchy prior. All inferences were based on posterior distributions generated using the Hamiltonian Markov Chain Monte Carlo (MCMC) method, and 95% credible intervals (CrI) were reported to quantify uncertainty (33). Considering interventions and study samples, within- or between-study heterogeneity in effect size estimates was assumed; therefore, a random-effects model was applied. In the meta-analysis, both within-study and between-study heterogeneity were quantified using τ (standard deviation). The potential scale reduction factor (PSRF) was used to assess model convergence and validity, with PSRF <1.01 indicating satisfactory convergence (34). The “true” effect size for each study was estimated using the ranef function and compared against the overall pooled effect. The Bayesian analysis results were reported by the BARG statement (see Supplementary Appendix 2). Forest plots were generated using the tidybayes and ggplot2 packages (R Programming Language, https://www.R-project.org/). Publication bias was evaluated by the assessment of funnel plot asymmetry, Egger's regression test, and Begg's rank test. Model convergence and posterior distributions are presented in Supplementary Appendix 5.

#### 2.6.2 Meta-regression

In this study, meta-regression models were constructed to explore the relationship between the effects of concurrent training (CT) on 6MWD and SGRQ. The influence of potential moderator variables, including participant age, body mass index (BMI), and the proportion of female participants, was also examined. Additionally, the influence of intervention duration on 6MWD and SGRQ scores was assessed through dose–response analyses.

#### 2.6.3 Dose–response meta-analysis

The 6MWD is a widely used test for objectively assessing exercise capacity in patients with moderate to severe COPD, demonstrating strong validity, reliability, and practical utility. In this relatively simple test, patients are instructed to walk as far as possible along a 30-m corridor for 6 min, with the primary outcome defined as the total distance covered. Previous studies have demonstrated that 6MWD serves not only as a measure of exercise capacity but also as a significant predictor of morbidity and mortality in individuals with COPD (35). Due to its strong correlation with VO_2max_ and clinical applicability, 6MWD is considered a surrogate gold standard for evaluating cardiorespiratory fitness (36). A Bayesian random-effects model was employed to analyze the dose–response relationship between CT and 6MWD. Based on the original studies, CT dose was operationalized as the product of exercise intensity [metabolic equivalents tasks (METs)] and weekly exercise duration, expressed in METs-min/week. Exercise intensity was coded to the 2024 Adult Physical Activity Guidelines and the ACSM Guidelines for Exercise Testing and Prescription (37). The MET values for aerobic and resistance training components in CT were independently coded per the above guidelines, and the combined mean MET value for the integrated CT program was calculated as the arithmetic average of the two individual MET values. A natural spline (four knots) was applied to capture the potential nonlinear association between CT dose and 6MWD. The predicted effects across different doses were presented with 95% Bayesian credible intervals (CrI) to reflect the estimation uncertainty, while heterogeneity was quantified using SD. The dose–response analysis was performed using the “brms” package in R (R Programming Language, https://www.R-project.org/). Data preprocessing and visualization were conducted using the “tidybayes” (R Programming Language, https://www.R-project.org/) and “ggplot2” packages. A CT training dose recommendation table was developed based on the 6MWD results.

## 3 Results

### 3.1 Study selection

**Figure 1:**
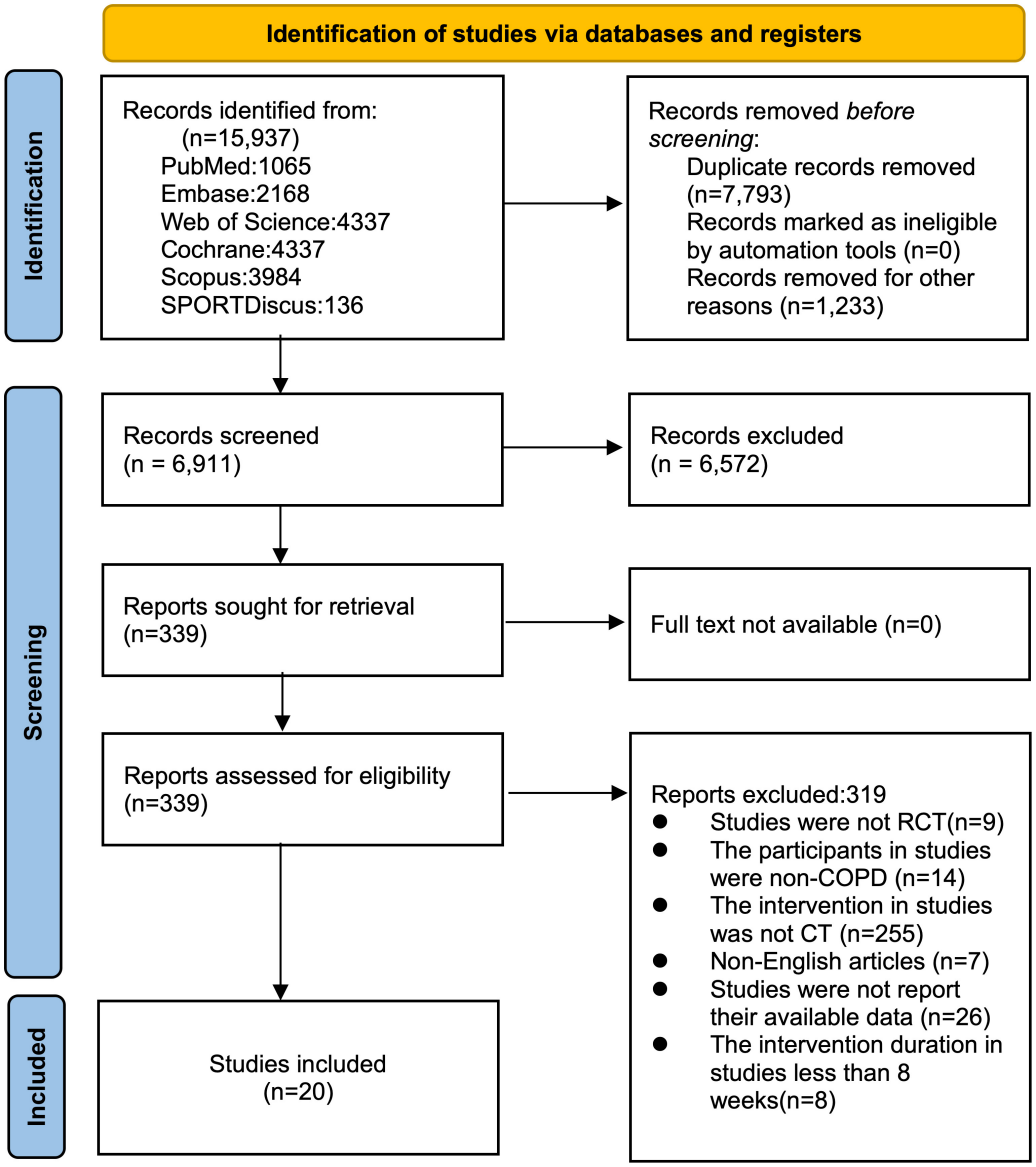
Literature review flowchart. COPD, chronic obstructive pulmonary disease; CT, concurrent training; RCT, randomized controlled trial.

A total of 15,937 studies were retrieved from six databases, including 1,065 from PubMed, 2,168 from Embase, 4,337 from Web of Science, 4,337 from the Cochrane Library, 3,894 from Scopus, and 136 from SPORTDiscus. After removing 7,793 duplicate studies, 6,911 studies were screened by title and abstract, resulting in 339 studies retained for full-text assessment. Following full-text screening, 20 randomized controlled trials (RCTs) were included in the meta-analysis (see Supplementary Appendix 4). Reasons for exclusion were as follows: inappropriate intervention population (*n* = 14), intervention protocol (*n* = 255), intervention duration (*n* = 8), study design (*n* = 9), unavailable outcome data (*n* = 26), and non-English studies (*n* = 7). See Figure 1 for the screening process.

### 3.2 Study characteristics

A total of 20 studies were included 1,037 participants (703 males and 334 females), with 538 in the CT group and 499 in the control group. The participants' mean age ranged from 58.4 to 79.8 years, and the mean BMI ranged from 21.5 to 32.5 kg/m^2^. The baseline 6MWD ranged from 147.5 to 504 m. Across the included studies, intervention duration ranged from 8 to 48 weeks, with training frequencies of 2–7 sessions per week and session lengths ranging from 45 to 150 min. Aerobic training modalities typically included cycling, running, walking, stair climbing, aerobic dance or calisthenics, while resistance training commonly involved chest press, leg press, dumbbell curls, and dumbbell shoulder press. Detailed characteristics of the included studies are provided in Supplementary Appendix 2.

### 3.3 Risk of bias and certainty of evidence

The Cochrane Risk of Bias 2 (RoB 2) tool was used to assess the risk of bias for each prespecified outcome measure in the 20 included studies individually, with the risk of bias varying by outcome within a single study. According to the GRADE system, the results indicated that the evidence quality for the 6MWD was rated as very low. The evidence quality for VO_2max_ and ESWT was rated as very low. The evidence for muscle strength (LP 1RM, CP 1RM, and Wpeak) and pulmonary function (FVC, FEV_1_, and FEV_1_/FVC) was also rated as very low. The evidence quality for quality of life (SGRQ) was rated as moderate. Overall, while CT provides benefits for certain outcomes in COPD patients, the quality of evidence remains low, and future research must address the existing limitations. Details of the GRADE assessment and risk of bias evaluation are provided in Supplementary Appendices 3 and 9.

### 3.4 Meta-analysis

#### 3.4.1 6MWD

**Figure 2:**
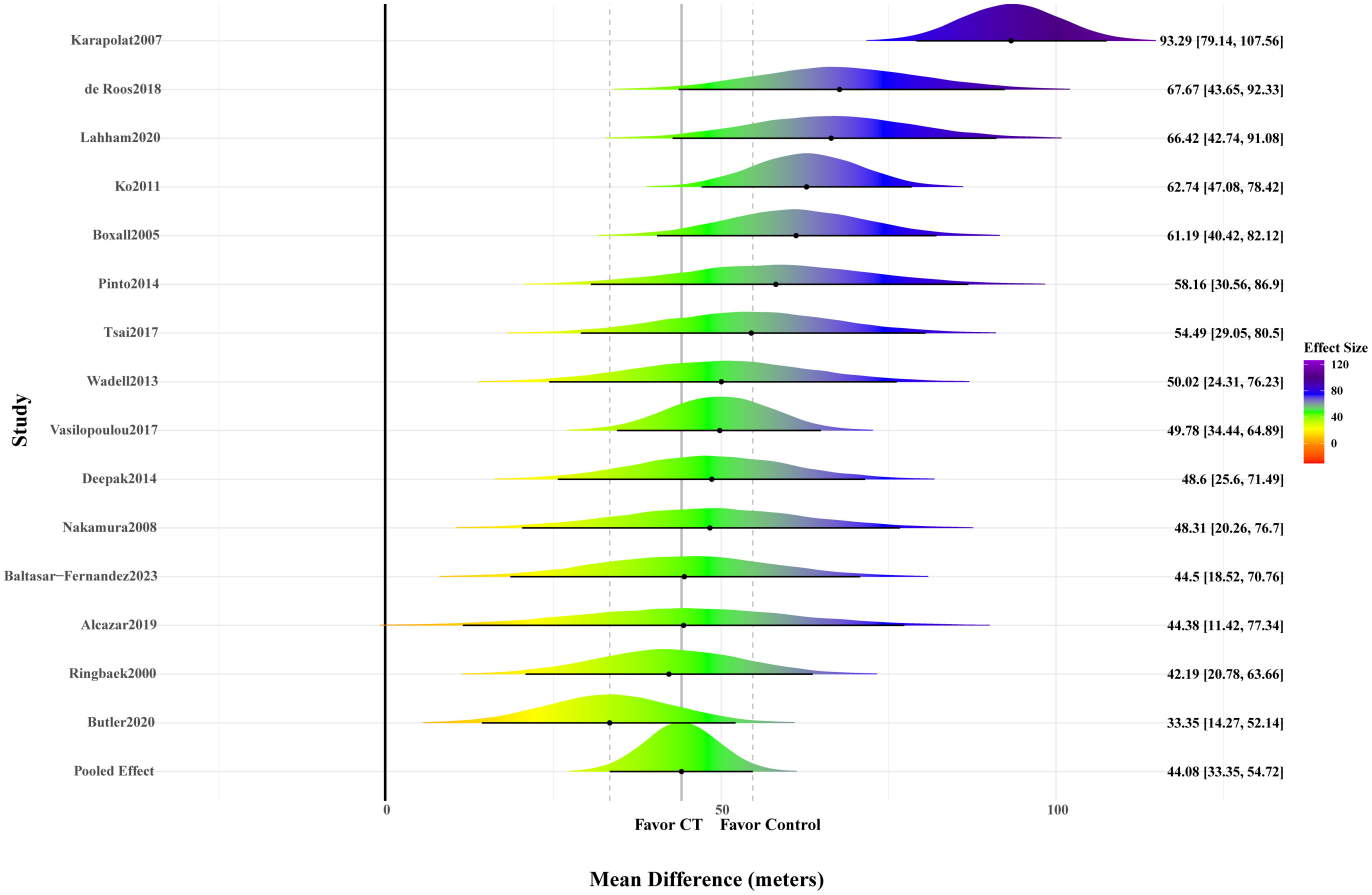
Forest plot of 6-minute walk distance (6MWD).

A total of 15 studies reporting 6MWD were included, comprising 755 participants (377 in the intervention group and 378 in the control group). The meta-analysis results indicated a significant improvement in 6MWD following CT among patients with COPD, with evidence of high heterogeneity and satisfactory model convergence (MD: 44.08; 95% CrI: 33.35–54.72; SD: 20.85; 95% CrI: 13.29–32.26; PSRF ≤ 1.01). Details are presented in Figure 2 and Table 1.

**Table 1:** Pairwise meta-analysis for all outcomes.

Outcomes	MD	SE	Lower 95% CrI	Upper 95% CrI	PSRF	SD (intercept)	SD-lower 95% CrI	SD-upper 95% CrI
6MWD	44.08	5.36	33.35	54.72	1.00	20.85	13.29	32.26
VO_2max_	1.02	0.50	0.04	2.00	1.00	0.91	0.23	2.12
ESWT	166.23	74.36	40.23	328.63	1.00	31.98	0.06	196.71
LP 1RM	30.53	13.82	3.38	57.71	1.00	2.52	0.04	15.19
CP 1RM	12.20	4.71	2.77	21.59	1.00	2.44	0.05	10.71
Wpeak	14.12	5.20	3.77	24.44	1.00	1.39	0.04	6.07
FVC	0.26	0.23	−0.19	0.71	1.00	0.79	0.02	3.06
FEV_1_	−0.28	1.82	−3.83	3.19	1.00	0.66	0.02	2.24
FEV_1_/FVC	−1.85	1.98	−5.69	2.00	1.00	0.83	0.03	3.10
SGRQ	−8.65	1.12	−10.79	−6.51	1.00	5.04	2.52	8.99

6MWD, 6-min walk distance; VO_2max_, maximal oxygen uptake; ESWT, endurance shuttle walk test; LP 1RM, leg press 1RM; CP 1RM, chest press 1RM; Wpeak, peak work rate; FVC, forced vital capacity; FEV_1_, forced expiratory volume in 1 s; FEV_1_/FVC, forced vital capacity/forced expiratory volume in 1 s; SGRQ, St. George's respiratory questionnaire. CT group (intervention): concurrent training; CG group (control): non-exercise intervention, usual care, wait-list control, or routine daily activities.

#### 3.4.2 VO_2*max*_

A total of five studies reporting VO_2max_ were included, comprising 184 participants (92 in each of the intervention and control groups). The meta-analysis results indicated a significant improvement in VO_2max_ following CT among patients with COPD, with evidence of high heterogeneity and satisfactory model convergence (MD: 1.02; 95% CrI: 0.04–2.00; SD: 0.91; 95% CrI: 0.23–2.12; PSRF ≤ 1.01). Details are presented in Supplementary Appendix 7 and Table 1.

#### 3.4.3 ESWT

A total of three studies reporting ESWT were included, comprising 126 participants (60 in the intervention group and 66 in the control group). The meta-analysis results indicated a significant improvement in ESWT following CT among patients with COPD, with evidence of moderate heterogeneity and satisfactory model convergence (MD: 166.23; 95% CrI: 40.23–328.63; SD: 31.98; 95% CrI: 0.06–196.71; PSRF ≤ 1.01). Details are presented in Supplementary Appendix 7 and Table 1.

#### 3.4.4 Leg press 1RM

A total of two studies reporting LP 1RM were included, comprising 43 participants (22 in the intervention group and 21 in the control group). The meta-analysis results indicated a significant improvement in LP 1RM following CT among patients with COPD, with evidence of high heterogeneity and satisfactory model convergence (MD: 30.53; 95% CrI: 3.38–57.71; SD: 2.52; 95% CrI: 0.04–15.19; PSRF ≤ 1.01). Details are presented in Supplementary Appendix 7 and Table 1.

#### 3.4.5 Chest press 1RM

A total of two studies reporting CP 1RM were included, comprising 43 participants (22 in the intervention group and 21 in the control group). The meta-analysis results indicated a significant improvement in CP 1RM following CT among patients with COPD, with evidence of moderate heterogeneity and satisfactory model convergence (MD: 12.2; 95% CrI: 2.77–21.59; SD: 2.44; 95% CrI: 0.05–10.71; PSRF ≤ 1.01). Details are presented in Supplementary Appendix 7 and Table 1.

#### 3.4.6 Wpeak

A total of four studies reporting Wpeak were included, comprising 140 participants (69 in the intervention group and 71 in the control group). The meta-analysis results indicated a significant improvement in Wpeak following CT among patients with COPD, with evidence of moderate heterogeneity and satisfactory model convergence (MD: 14.12; 95% CrI: 3.77–24.44; SD: 1.39; 95% CrI: 0.04–6.07; PSRF ≤ 1.01). Details are presented in Supplementary Appendix 7 and Table 1.

#### 3.4.7 FVC

A total of four studies reporting FVC were included, comprising 153 participants (79 in the intervention group and 74 in the control group). The meta-analysis results indicated no significant improvement in FVC following CT among patients with COPD, with evidence of high heterogeneity and satisfactory model convergence (MD: 0.26; 95% CrI: −0.19 to 0.71; SD: 0.79; 95% CrI: 0.02–3.06; PSRF ≤ 1.01). Details are presented in Supplementary Appendix 7 and Table 1.

#### 3.4.8 FEV_1_

A total of five studies reporting FEV_1_ were included, comprising 213 participants (109 in the intervention group and 104 in the control group). The meta-analysis results indicated no significant improvement in FEV_1_ following CT among patients with COPD, with evidence of moderate heterogeneity and satisfactory model convergence (MD: −0.28; 95% CrI: −3.83 to 3.19; SD: 0.66; 95% CrI: 0.02–2.24; PSRF ≤ 1.01). Details are presented in Supplementary Appendix 7 and Table 1.

#### 3.4.9 FEV_1_/FVC

A total of three studies reporting FEV_1_/FVC were included, comprising 99 participants (49 in the intervention group and 50 in the control group). The meta-analysis results indicated no significant improvement in FEV_1_/FVC following CT among patients with COPD, with evidence of moderate heterogeneity and satisfactory model convergence (MD: −1.85; 95% CrI: −5.69 to 2.00; SD: 0.83; 95% CrI: 0.03–3.10; PSRF ≤ 1.01). Details are presented in Supplementary Appendix 7 and Table 1.

#### 3.4.10 SGRQ

**Figure 3:**
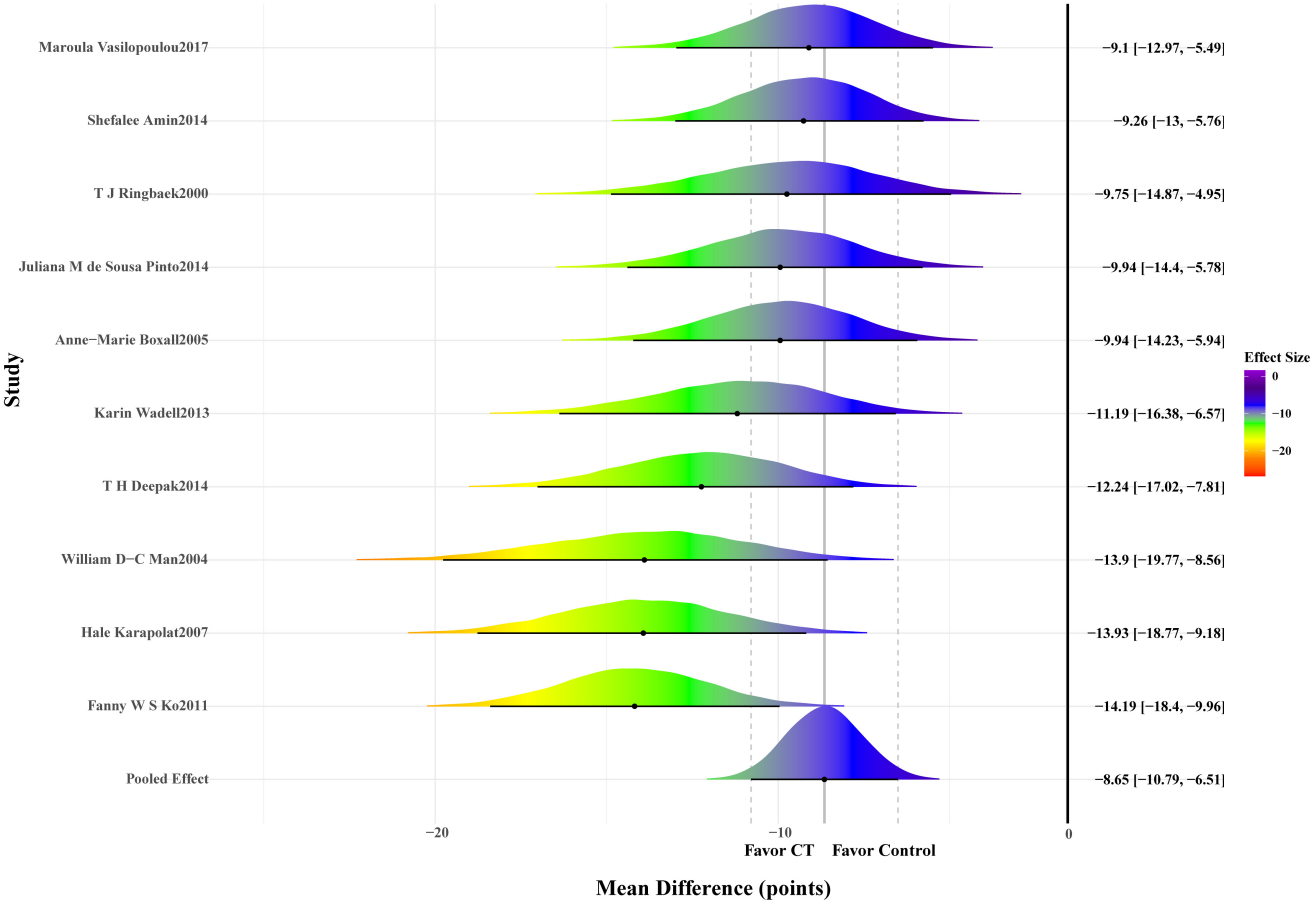
Forest plot of St. George's Respiratory Questionnaire (SGRQ).

A total of 10 studies reporting the SGRQ total score were included, comprising 503 participants (252 in the intervention group and 251 in the control group). The meta-analysis results indicated a significant improvement in SGRQ total scores following CT among patients with COPD, thereby improving their quality of life, with evidence of high heterogeneity and satisfactory model convergence (MD: −8.65; 95% CrI: −10.79 to −6.51; SD: 5.04; 95% CrI: 2.52–8.99; PSRF ≤ 1.01). Details are presented in Figure 3 and Table 1.

Among the 10 outcome indicators, only 6MWD and SGRQ total score were assessed in more than 10 studies; the remaining indicators were reported in fewer than 10 studies, and thus the results of the publication bias assessment should be interpreted with caution. According to Egger's and Begg's tests, evidence of publication bias was found exclusively for 6MWD (Egger's test ≤ 0.05; Begg's test ≤ 0.05). See Supplementary Appendix 4 for the funnel plot results assessing publication bias.

### 3.5 Dose–response meta analysis

**Figure 4:**
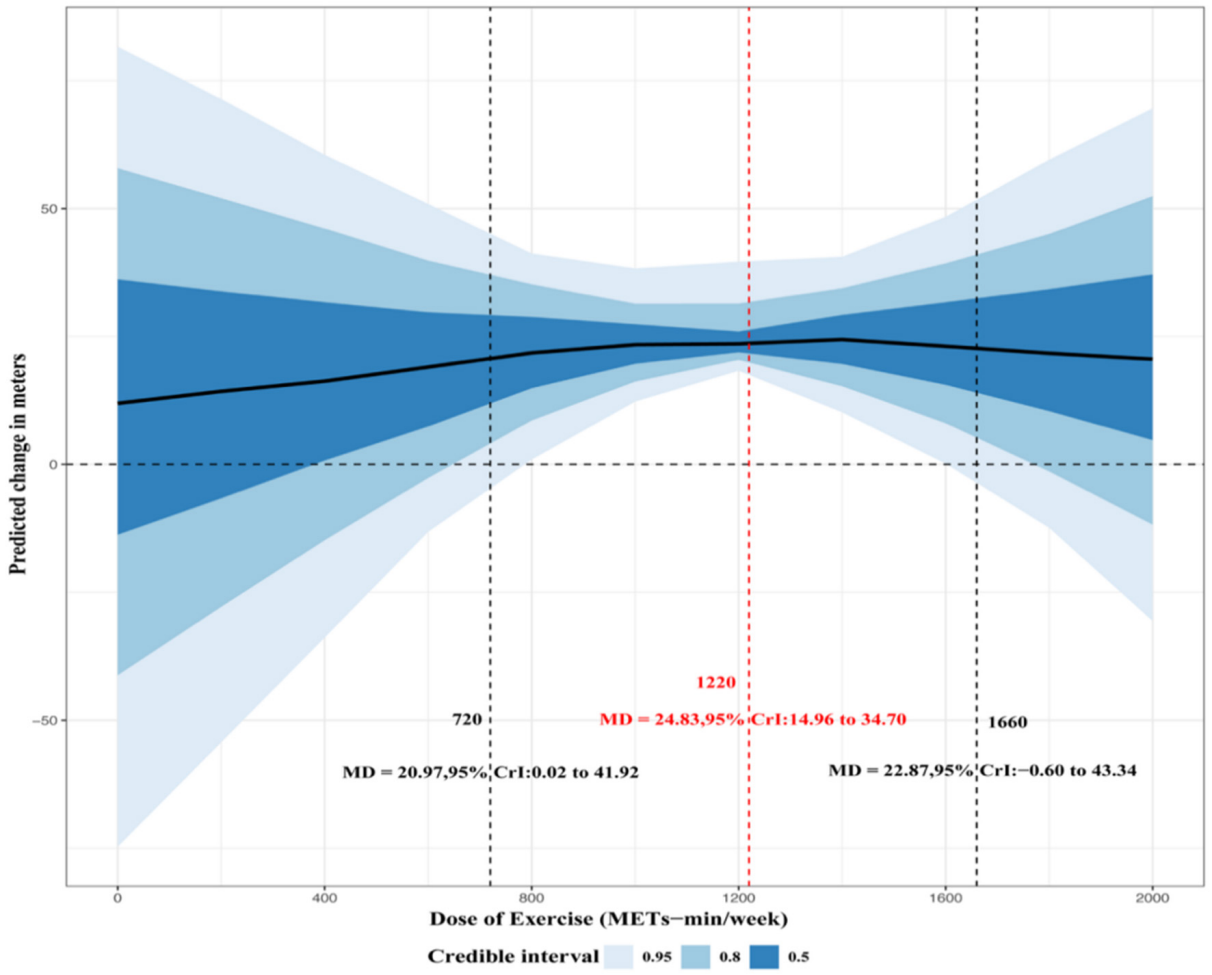
Dose–response relationship between concurrent training and 6-min walk distance (6MWD).

The dose-response relationship between CT and 6MWD is illustrated in Figure 4. CT and 6MWD were characterized by a nonlinear dose–response relationship, indicating that increasing the exercise dose does not necessarily yield proportionally greater improvements. Beyond a certain threshold, further increases in exercise volume were associated with limited additional benefits or even a slight decline in effectiveness. The estimated minimum effective dose for improving 6MWD was 720 MET min/week (MD = 20.97; 95% CrI: 0.02–41.92), while the optimal dose was 1,220 MET-min/week (MD = 24.83; 95% CrI: 14.96–34.70), and the maximum tolerated dose was 1,660 MET-min/week (MD = 22.87; 95% CrI: −0.60 to 43.34). Additionally, practical recommendations based on the estimated minimal clinically important dose and optimal doses of CT are provided in Table 2. A detailed presentation of the estimated dose-response relationship is available in Supplementary Appendix 6.

**Table 2:** CT dose recommendation.

CT dose^a^ (METs-min/week)	Intensity		Energy expenditure^b^ (METs-min)	Recommended accumulation^c^ (min/week)	Recommendations for exercise prescription^d^ (sessions × min/per week)	
Minimal clinically important dose	720	Moderate	3.5 (01018, 02054)	205	3 × 68	4 × 51
			4.0 (01010, 02054)	180	3 × 60	2 × 90
			5.0 (17032, 02052)	144	3 × 48	2 × 72
		Vigorous	6.5 (02057, 17032)	110	3 × 37	2 × 55
			7.0 (01016, 02057)	102	3 × 34	2 × 51
			8.0 (02057, 12030)	90	2 × 45	1 × 90
Optimal dose	1,220	Moderate	3.5 (01018, 02054)	350	5 × 70	6 × 58
			4.0 (01010, 02054)	305	5 × 61	6 × 51
			5.0 (17032, 02052)	244	5 × 49	6 × 41
		Vigorous	6.5 (02057, 17032)	188	4 × 47	5 × 38
			7.0 (01016, 02057)	174	4 × 44	5 × 35
			8.0 (02057, 12030)	153	4 × 38	5 × 30

CT, concurrent training.

^a^Dose thresholds were established based on the dose–response meta-analysis results derived from the current study.

^b^Intensity coding was extracted from the Compendium of Physical Activity: Code 01018: Bicycling, leisure 5.5 mph; Code 02054: Resistance (weight) training, multiple exercises, 8–15 reps at varied resistance; Code 01010: Bicycling, <10 mph, leisure, to work or for pleasure (Taylor Code 115); Code 17032: Climbing hills, no load, 5%−20% grade, very slow pace; Code 02052: Resistance (weight) training, squats, deadlift, slow or explosive effort; Code 02057: Body weight resistance exercises (e.g., squat, lunge, push-up, crunch), high intensity; Code 01016: Bicycling, self-selected moderate pace; Code 12030: Running, 5.0–5.2 mph (12 min/mile).

^c^Minimum weekly time of exercise.

^d^Frequency and duration of each exercise, not counting warm-up and cool-down.

## 4 Discussion

This study is the first meta-analysis to comprehensively describe the effects of CT on patients with COPD, systematically evaluating the intervention effects of CT on exercise capacity and quality of life. Compared with the control group, CT significantly improved patients' 6MWD, VO_2max_, ESWT, Wpeak, and other related indicators, whereas no significant changes were observed in pulmonary function indicators including FVC, FEV_1_, and FEV_1_/FVC. In addition, we found that the optimal intervention effect on 6MWD was achieved when the exercise dose reached 1,220 MET-min/week. Furthermore, the intervention effects of CT may be moderated by factors such as age, with older age being associated with weaker improvements in 6MWD.

### 4.1 The effect of concurrent training on cardiopulmonary fitness

6MWD, VO_2max_, and ESWT are important indicators for evaluating cardiorespiratory fitness in patients with COPD (38–40) and have been identified as key predictors of mortality in this population. In this study, a multilevel Bayesian meta-analysis was conducted to compare differences in cardiorespiratory function between patients with COPD undergoing CT and those without training. The results showed that CT significantly improved 6MWD and demonstrated a tendency to improve VO_2max_ and ESWT, suggesting that CT may exert beneficial effects on cardiorespiratory fitness in patients with COPD. Several previous studies have also confirmed the potential benefits of CT in improving cardiorespiratory fitness in patients with COPD (41). The mechanisms through which CT improves cardiorespiratory fitness in patients with COPD may involve several aspects. First, CT may enhance pulmonary ventilation and gas exchange capacity and increase the ability of the blood and circulatory system to transport oxygen, thereby improving overall oxygen transport efficiency. Second, CT may activate signaling pathways such as AMPK/PGC-1α, promoting mitochondrial biogenesis and capillary formation in skeletal muscle and consequently improving skeletal muscle oxygen utilization capacity (42). Third, CT may enhance lower-limb muscle strength and tendon stiffness, thereby improving neuromuscular control and increasing walking efficiency and exercise tolerance (43). Therefore, CT may comprehensively improve cardiorespiratory fitness in patients with COPD by enhancing oxygen transport, oxygen utilization efficiency, and neuromuscular control. In addition, the present study found a dose-response relationship between CT and 6MWD. Xie et al. (44) reported a dose-response relationship between exercise intensity and 6MWD, suggesting that the optimal dose ranged from 350 to 620 MET-min/week. The present study further demonstrated that the optimal dose of CT for improving 6MWD was 1,220 MET-min/week, and that a clear improvement trend appeared when the exercise dose reached 720 MET-min/week. This threshold is slightly higher than the minimum weekly physical activity recommendation of 600 MET-min/week proposed by the World Health Organization, which may be because this study focused on CT as a specific exercise modality that integrates both aerobic and resistance training components and may therefore produce more complex and comprehensive physiological stimuli. The meta-regression results further showed that age was significantly negatively associated with 6MWD, indicating that the intervention effect of CT on 6MWD weakened with increasing age. This may be related to age-associated reductions in maximal cardiac output, pulmonary function, and skeletal muscle oxygen transport capacity (45). Although CT also showed positive effects on VO_2max_ and ESWT, the results should be interpreted with caution due to the limited number of available studies. Details are presented in Supplementary Appendix 8.

### 4.2 The effect of concurrent training on muscle strength

Wpeak is commonly used as an indirect indicator of muscle function, whereas LP 1RM and CP 1RM can directly assess maximal muscle strength (46). The results of the present study showed that CT could improve Wpeak, LP 1RM, and CP 1RM to a certain extent in patients with COPD, which is generally consistent with previous studies (47, 48). Some studies have suggested that the potential mechanisms underlying improvements in neuromuscular adaptations may involve enhanced neural activation, increased muscle fiber pennation angle, and changes in fascicle length. In addition, improvements in maximal strength may be related to the activation of the Akt–mTORC1 signaling pathway induced by resistance training, which promotes muscle protein synthesis and induces muscle fiber hypertrophy, thereby increasing maximal muscle strength (49–51). Previous studies have also indicated that muscle strength in patients with COPD may be influenced by sex-related factors. In terms of sex differences, male patients are more likely to exhibit certain limitations in lower-limb maximal muscle strength, whereas obvious inhibitory responses are less frequently observed in female patients. This may be related to the higher proportion of type II muscle fibers in males, which are more susceptible to fatigue, as well as greater fluctuations in testosterone and cortisol levels. In contrast, females tend to have a higher proportion of type I muscle fibers, and estrogen may exert protective effects in maintaining muscle function and reducing muscle damage (52). In the present study, the number of included studies reporting muscle strength outcomes was relatively small (LP 1RM and CP 1RM: two studies; Wpeak: four studies); therefore, these findings should be interpreted with caution and require further confirmation from additional high-quality studies.

### 4.3 The effect of concurrent training on quality of life

The SGRQ is a commonly used questionnaire in clinical practice with good reliability and validity for comprehensively assessing the health status of patients with COPD, with lower scores indicating better quality of life (53, 54). COPD is a disease characterized by recurrent exacerbations and progressive deterioration, typically manifested by gradual declines in cardiorespiratory fitness and exercise capacity, often accompanied by dyspnea, psychological disorders, and malnutrition, all of which severely impair patients' quality of life (55). The results of the present study showed that CT could reduce the total SGRQ score in patients with COPD, thereby improving quality of life to a certain extent, which is consistent with previous studies (48). Some studies have suggested that this improvement may be related to the ability of CT to alleviate negative emotions such as tension, anxiety, and depression, thereby enhancing patients' confidence in treatment. In addition, CT may further improve quality of life by increasing muscle strength and enhancing the ability to perform daily activities (56).

The meta-regression results indicated that BMI was negatively associated with changes in SGRQ scores, suggesting that patients with higher BMI experienced greater reductions in SGRQ scores after the intervention. This finding may be related to the presence of more anti-inflammatory factors in overweight or obese patients with COPD (57). For example, lipoproteins can bind to and neutralize endotoxins, thereby exerting anti-inflammatory effects and potentially improving patients' quality of life. To some extent, this finding suggests the presence of an “obesity paradox” between BMI and quality of life (58). Future studies are warranted to further explore the potential mechanisms through which BMI and other factors influence improvements in quality of life among patients with COPD. Details are presented in Supplementary Appendix 8.

### 4.4 The effect of concurrent training on pulmonary function

FVC, FEV_1_, and FEV_1_/FVC are important indicators for evaluating pulmonary function. They are not only widely used to assess the severity of COPD but are also closely associated with patient survival (59). Due to the irreversible structural alterations in the airways of patients with COPD, pulmonary function is typically markedly reduced compared with healthy individuals (60). The results of the present study showed that CT did not produce significant improvements in FVC, FEV_1_, or FEV_1_/FVC, which is generally consistent with findings from previous studies (61). Previous research has suggested that this phenomenon may be attributed to the core pathological characteristics of COPD, including irreversible airway narrowing, alveolar destruction, and reduced elastic recoil of the lungs. Although exercise interventions may improve overall health status in patients with COPD, their capacity to reverse structural damage to lung tissue is likely limited (61).

It is worth noting that mind–body exercise interventions such as Qigong may exert certain positive effects on pulmonary function in patients with COPD, for example by improving FVC and alleviating symptoms of dyspnea (44). This finding suggests that different exercise modalities may exert distinct effects on pulmonary function indicators, and the underlying mechanisms require further investigation. Given the limited effects of CT on pulmonary function observed in the present study, future research may explore the potential synergistic effects of combining CT with other interventions (e.g., inspiratory muscle training) to achieve greater benefits in improving pulmonary function (62, 63).

## 5 Clinical implications

The present study provides several important clinical implications. First, the findings confirm that CT can significantly improve 6MWD in patients with COPD and may also enhance exercise performance indicators such as VO_2max_, ESWT, and Wpeak to a certain extent, while exerting positive effects on patients' quality of life. Second, previous studies have not clearly identified the optimal exercise dose of CT for patients with COPD. In the present study, dose-response analysis estimated that the potential optimal dose of CT for improving 6MWD in patients with COPD was approximately 1,220 MET-min/week, which is equivalent to about 244 min of moderate-intensity exercise or 153 min of vigorous-intensity exercise per week, and the minimum effective dose (720 MET-min/week) was also provided as a reference for clinical practice. This dose-response relationship offers key evidence for clinicians when developing individualized exercise prescriptions, thereby ensuring intervention effectiveness while avoiding insufficient training stimulus or excessive burden, and improving exercise safety and feasibility.

Our findings also highlight the importance of considering individual differences when implementing CT interventions. For example, age was significantly negatively associated with improvements in 6MWD, whereas patients with higher BMI tended to exhibit greater improvements in SGRQ. Therefore, when designing CT intervention programs, exercise prescriptions should be individualized according to patient characteristics such as age and BMI. In addition, regular exercise monitoring should be conducted during the intervention period so that training plans can be adjusted in a timely manner according to patients' adaptation, thereby improving the precision and effectiveness of exercise interventions.

## 6 Strengths and limitations

The present study has several strengths. First, the application of Bayesian methods enabled the integration of experimental data from different studies, thereby enhancing the accuracy and robustness of the analytical results (64). In dealing with study heterogeneity, Bayesian approaches offer certain advantages, allowing more precise estimation of the magnitude of heterogeneity and its potential sources, such as differences in study design or sample characteristics. In addition, this study incorporated a natural spline–based dose–response model into the meta-analysis framework. This approach not only evaluated the overall intervention effects of CT but also further explored differences related to exercise dose and intervention duration. Such an analytical framework may facilitate the development of more scientifically grounded exercise prescriptions and provide a theoretical basis for individualized interventions in clinical practice, particularly for patients with COPD.

However, several limitations should also be acknowledged. First, the overall quality of the included studies was relatively low, and some randomized controlled trials did not adequately implement blinding procedures, which may have affected the overall quality of the evidence. Second, the number of included studies remained limited, and some key outcomes (e.g., VO_2max_, ESWT, FVC, and SGRQ) could not be further analyzed in greater depth, thereby restricting the comprehensiveness of the evaluation of CT effects. Third, the original studies included in this meta-analysis compared CT only with usual care or non-exercise control groups, rather than directly comparing CT with aerobic training alone or resistance training alone. Therefore, the potential interaction or interference effects between different training modalities could not be fully examined. Moreover, most of the included randomized controlled trials reported the overall training dose of CT but did not clearly specify the sequence of the two training components (i.e., whether aerobic training was performed before or after resistance training), although training sequence may potentially influence intervention outcomes. Finally, due to limitations in the original literature, the meta-regression analyses did not account for other potentially relevant factors, such as supervision during training or funding support, which may have affected the completeness of the results.

## 7 Conclusions

The present study employed a multilevel Bayesian pairwise and dose–response meta-analysis to systematically evaluate the intervention effects of CT in patients with COPD. The results showed that CT significantly improved 6MWD and demonstrated beneficial effects on VO_2max_, ESWT, Wpeak, LP 1RM, CP 1RM, and SGRQ to a certain extent, suggesting that CT may exert positive effects on exercise capacity and quality of life in patients with COPD. In addition, a nonlinear dose–response relationship was observed between CT and 6MWD. The findings indicated that performing approximately 244 min of moderate-intensity CT or 153 min of vigorous-intensity CT per week may achieve better intervention effects. Further analyses showed that younger patients with COPD exhibited greater improvements in 6MWD, whereas patients with higher BMI tended to demonstrate more pronounced improvements in SGRQ.

## Data Availability

The original contributions presented in the study are included in the article/Supplementary material, further inquiries can be directed to the corresponding author.
