# Supplementary material for: Effects of concurrent training on exercise capacity and quality of life in older adult patients with COPD: a Bayesian pairwise and dose–response meta-analysis
Source: Front Med (Lausanne). 2026 Apr 9;13:1760792. doi: 10.3389/fmed.2026.1760792 (PMC13102676; doi:10.3389/fmed.2026.1760792)
Supplement: Supplementary file 2 [file Supplementary_file_2.docx]

**Effects of Concurrent Training on Exercise Capacity and Quality of Life in Elderly Patients with COPD: A Bayesian Pairwise and Dose–Response Meta-Analysis**

**Appendix**

**Contents**

[1.Search strategy 2](#_Toc1377544543)

[1.1Pubmed 2](#_Toc369935894)

[1.2Embase 3](#_Toc547412393)

[1.3Web of Science 4](#_Toc540145403)

[1.4Cochrane Library 5](#_Toc810412352)

[1.5Scopus 6](#_Toc1259110790)

[1.6SPORTDiscus 6](#_Toc571189992)

[2.Demographic characteristics of included studies 7](#_Toc738293454)

[3.Risk of bias 9](#_Toc337569012)

[4.Publication bias assessments 10](#_Toc2018072957)

[5.Convergence and posterior distributions 15](#_Toc395467581)

[6.Predicted dose-responses 18](#_Toc161746402)

[7.Forest plot for pairwise comparison 20](#_Toc1904964959)

[8.Meta‑regression 24](#_Toc2059856437)

[9.GRADE Evidence Quality Rating 27](#_Toc423263372)

[10.Included studies 30](#_Toc1321654340)

# 1.Search strategy

## 1.1Pubmed

|  | Search Details | Results |
| --- | --- | --- |
| #1 | ((("Pulmonary Disease, Chronic Obstructive"[Mesh]) OR ((((((((((Chronic Obstructive Pulmonary Diseases[Title/Abstract]) OR (COPD[Title/Abstract])) OR (Chronic Obstructive Lung Disease[Title/Abstract])) OR (Chronic Obstructive Pulmonary Disease[Title/Abstract])) OR (COAD[Title/Abstract])) OR (Chronic Obstructive Airway Disease[Title/Abstract])) OR (Airflow Obstruction, Chronic[Title/Abstract])) OR (Airflow Obstructions, Chronic[Title/Abstract])) OR (Chronic Airflow Obstructions[Title/Abstract])) OR (Chronic Airflow Obstruction[Title/Abstract]))) | 117665 |
| #2 | (("Exercise"[Mesh]) OR (((((((((((((((((((((((((Exercises[Title/Abstract]) OR (Exercise, Physical[Title/Abstract])) OR (Exercises, Physical[Title/Abstract])) OR (Physical Exercise[Title/Abstract])) OR (Physical Exercises[Title/Abstract])) OR (Physical Activity[Title/Abstract])) OR (Activities, Physical[Title/Abstract])) OR (Activity, Physical[Title/Abstract])) OR (Physical Activities[Title/Abstract])) OR (Exercise, Aerobic[Title/Abstract])) OR (Aerobic Exercise[Title/Abstract])) OR (Aerobic Exercises[Title/Abstract])) OR (Exercises, Aerobic[Title/Abstract])) OR (Exercise, Isometric[Title/Abstract])) OR (Exercises, Isometric[Title/Abstract])) OR (Isometric Exercises[Title/Abstract])) OR (Isometric Exercise[Title/Abstract])) OR (Acute Exercise[Title/Abstract])) OR (Acute Exercises[Title/Abstract])) OR (Exercise, Acute[Title/Abstract])) OR (Exercises, Acute[Title/Abstract])) OR (Exercise Training[Title/Abstract])) OR (Exercise Trainings[Title/Abstract])) OR (Training, Exercise[Title/Abstract])) OR (Trainings, Exercise[Title/Abstract])))) | 484713 |
| #3 | (((Randomized controlled trial[Title/Abstract]) OR (Randomized[Title/Abstract])) OR (Placebo[Title/Abstract])) | 898808 |
| #4 | #1 AND #2 AND #3 | 1065 |

## 1.2Embase

|  | Search Details | Results |
| --- | --- | --- |
| #1 | 'Chronic Obstructive Pulmonary Diseases':ab,ti OR 'COPD':ab,ti OR 'Chronic Obstructive Pulmonary Disease':ab,ti OR 'COAD':ab,ti OR 'Chronic Obstructive Airway Disease':ab,ti OR 'Airflow Obstruction, Chronic':ab,ti OR 'Airflow Obstructions, Chronic':ab,ti OR 'Chronic Airflow Obstructions':ab,ti OR 'Chronic Airflow Obstruction':ab,ti OR 'Pulmonary Disease, Chronic Obstructive':ab,ti | 223284 |
| #2 | 'Exercises':ab,ti OR 'Exercise, Physical':ab,ti OR 'Exercises, Physical':ab,ti OR 'Physical Exercise':ab,ti OR 'Physical Exercises':ab,ti OR 'Physical Activity':ab,ti OR 'Activities, Physical':ab,ti OR 'Activity, Physical':ab,ti OR 'Physical Activities':ab,ti OR 'Exercise, Aerobic':ab,ti OR 'Aerobic Exercise':ab,ti OR 'Aerobic Exercises':ab,ti OR 'Exercises, Aerobic':ab,ti OR 'Exercise, Isometric':ab,ti OR 'Exercises, Isometric':ab,ti OR 'Isometric Exercises':ab,ti OR 'Isometric Exercise':ab,ti OR 'Acute Exercise':ab,ti OR 'Acute Exercises':ab,ti OR 'Exercise, Acute':ab,ti OR 'Exercises, Acute':ab,ti OR 'Exercise Training':ab,ti OR 'Exercise Trainings':ab,ti OR 'Training, Exercise':ab,ti OR 'Trainings, Exercise':ab,ti | 679822 |
| #3 | 'Randomized controlled trial':ab,ti OR 'Randomized':ab,ti OR 'Placebo':ab,ti | 1235785 |
| #4 | #1 AND #2 AND #3 | 2168 |

## 1.3Web of Science

|  | Search Details | Results |
| --- | --- | --- |
| #1 | Pulmonary Disease, Chronic Obstructive (Topic) or Chronic Obstructive Pulmonary Diseases (Topic) or COPD (Topic) or Chronic Obstructive Lung Disease (Topic) or Chronic Obstructive Pulmonary Disease (Topic) or COAD (Topic) or Chronic Obstructive Airway Disease (Topic) or Airflow Obstruction, Chronic (Topic) or Airflow Obstructions, Chronic (Topic) or Chronic Airflow Obstructions (Topic) or Chronic Airflow Obstruction (Topic) | 171451 |
| #2 | Exercise (Topic) or Exercises (Topic) or Exercise, Physical (Topic) or Exercises, Physical (Topic) or Physical Exercise (Topic) or Physical Exercises (Topic) or Physical Activity (Topic) or Activities, Physical(Topic) or Activity, Physical (Topic) or Physical Activities (Topic) or Exercise, Aerobic (Topic) or Aerobic Exercise (Topic) or Aerobic Exercises (Topic) or Exercises, Aerobic (Topic) or Exercise, Isometric (Topic) or Exercises, Isometric (Topic) or Isometric Exercises (Topic) or Isometric Exercise (Topic) or Acute Exercise(Topic) or Acute Exercises (Topic) or Exercise, Acute (Topic) or Exercises, Acute (Topic) or Exercise Training (Topic) or Exercise Trainings (Topic) or Training, Exercise (Topic) or Trainings, Exercise (Topic) | 1841252 |
| #3 | Randomized controlled trial (Topic) or Randomized (Topic) or Placebo (Topic) or Randomised (Topic) or random (Topic) or rct (Topic) | 2651216 |
| #4 | #1 AND #2 AND #3 | 4337 |

## 1.4Cochrane Library

|  | Search Details | Results |
| --- | --- | --- |
| #1 | (Chronic Obstructive Pulmonary Diseases):ti,ab,kw OR (COPD):ti,ab,kw OR (Chronic Obstructive Lung Disease):ti,ab,kw OR (Chronic Obstructive Pulmonary Disease):ti,ab,kw OR (COAD):ti,ab,kw OR (Chronic Obstructive Airway Disease):ti,ab,kw OR (Airflow Obstruction, Chronic):ti,ab,kw OR (Airflow Obstructions, Chronic):ti,ab,kw OR (Chronic Airflow Obstructions):ti,ab,kw OR (Chronic Airflow Obstruction):ti,ab,kw OR (Pulmonary Disease, Chronic Obstructive):ti,ab,kw | 26031 |
| #2 | (Exercise):ti,ab,kw OR (Exercises):ti,ab,kw OR (Exercise, Physical):ti,ab,kw OR (Exercises, Physical):ti,ab,kw OR (Physical Exercise):ti,ab,kw OR (Physical Exercises):ti,ab,kw OR (Physical Activity):ti,ab,kw OR (Activities, Physical):ti,ab,kw OR (Activity, Physical):ti,ab,kw OR (Physical Activities):ti,ab,kw OR (Exercise, Aerobic):ti,ab,kw OR (Aerobic Exercise):ti,ab,kw OR (Aerobic Exercises):ti,ab,kw OR (Exercises, Aerobic):ti,ab,kw OR (Exercise, Isometric):ti,ab,kw OR (Exercises, Isometric):ti,ab,kw OR (Isometric Exercises):ti,ab,kw OR (Isometric Exercise):ti,ab,kw OR (Acute Exercise):ti,ab,kw OR (Acute Exercises):ti,ab,kw OR (Exercise, Acute):ti,ab,kw OR (Exercises, Acute):ti,ab,kw OR (Exercise Training):ti,ab,kw OR (Exercise Trainings):ti,ab,kw OR (Training, Exercise):ti,ab,kw OR (Trainings, Exercise):ti,ab,kw | 150114 |
| #3 | #1 AND #2 | 4337 |

## 1.5Scopus

|  | Search Details | Results |
| --- | --- | --- |
| #1 | ( TITLE-ABS-KEY ( "exercise" OR "exercises" OR "exercise, physical" OR "exercises, physical" OR "physical exercise" OR "physical exercises" OR "physical activity" OR "activities, physical" OR "activity, physical" OR"physical activities" OR "exercise, aerobic" OR "aerobic exercise" OR "aerobic exercises" OR "exercises, aerobic" OR "exercise, isometric" OR "exercises, isometric" OR "isometric exercises" OR "isometric exercise"OR "acute exercise" OR "acute exercises" OR "exercise, acute" OR "exercises, acute" OR "exercise training"OR "exercise trainings" OR "training, exercise" OR "trainings, exercise" ) AND TITLE-ABS-KEY ( "pulmonary disease, chronic obstructive" OR "chronic obstructive pulmonary diseases" OR "copd" OR "chronic obstructive lung disease" OR "chronic obstructive pulmonary disease" OR "coad" OR "chronic obstructive airway disease" OR "airflow obstruction, chronic" OR "airflow obstructions, chronic" OR "chronic airflow obstructions" OR "chronic airflow obstruction" ) AND TITLE-ABS-KEY ( "randomized controlled trial" OR"randomized" OR "placebo" ) ) | 3894 |

## 1.6SPORTDiscus

|  | Search Details | Results |
| --- | --- | --- |
| #1 | (Exercise OR Exercises OR Exercise, Physical OR Exercises, Physical OR Physical Exercise OR Physical Exercises OR Physical Activity OR Activities, Physical OR Activity, Physical OR Physical Activities OR Exercise, Aerobic OR Aerobic Exercise OR Aerobic Exercises OR Exercises, Aerobic OR Exercise, Isometric OR Exercises, Isometric OR Isometric Exercises OR Isometric Exercise OR Acute Exercise OR Acute Exercises OR Exercise, Acute OR Exercises, Acute OR Exercise Training OR Exercise Trainings OR Training, Exercise OR Trainings, Exercise) AND (Pulmonary Disease, Chronic Obstructive OR Chronic Obstructive Pulmonary Diseases OR COPD OR Chronic Obstructive Lung Disease OR Chronic Obstructive Pulmonary Disease OR COAD OR Chronic Obstructive Airway Disease OR Airflow Obstruction, Chronic OR Airflow Obstructions, Chronic OR Chronic Airflow Obstructions OR Chronic Airflow Obstruction) AND (Randomized controlled trial OR Randomized OR Placebo) | 136 |

# 2.Demographic characteristics of included studies

| First Author  (Year) | Country | Intervention Type | Sample Size (Male) | Age (Years) | Week | Frequency (Sessions/Week) | METs  （Session） | METs  （Week） | Outcomes | Follow-Up (Yes/No) |
| --- | --- | --- | --- | --- | --- | --- | --- | --- | --- | --- |
| Alcazar2019 | Spain | CT | 14(11) | 77.7±7.9 | 12 | 2 | 5.9 | 590 | ①②⑥⑦⑧⑨ | N |
|  |  | CG | 15(13) | 79.8±6.4 |  |  |  |  |  |  |
| Amin2014 | USA | CT | 9(3) | 66.8±8.1 | 12 | 2 | 4.75 | 712.5 | ⑩ | N |
|  |  | CG | 10(6) | 72.0±10.1 |  |  |  |  |  |  |
| Baltasar-Fernandez2023 | Spain | CT | 8(5) | 73.6±6.3 | 12 | 2 | 6.75 | 810 | ①②④⑤⑥⑦⑧⑨ | Y |
|  |  | CG | 13(12) | 79.0±6.4 |  |  |  |  |  |  |
| Boxall2005 | Australia | CT | 23(11) | 77.6±7.6 | 12 | 7 | 2.9 | 1218 | ①⑩ | N |
|  |  | CG | 23(15) | 75.8±8.1 |  |  |  |  |  |  |
| Butler2020 | Canada | CT | 49(28) | 68±9 | 48 | 2 | 2.3 | 414 | ① | Y |
|  |  | CG | 48(22) | 69±9 |  |  |  |  |  |  |
| de Roos2018 | Netherlands | CT | 26(8) | 69.4±9.7 | 10 | 2 | 6.9 | 828 | ① | N |
|  |  | CG | 26(10) | 71±9.4 |  |  |  |  |  |  |
| Pinto2014 | Spain | CT | 23(22) | 68.9±9.2 | 12 | 2 | 4.17 | 375.3 | ①⑩ | N |
|  |  | CG | 18(17) | 71.9±7.6 |  |  |  |  |  |  |
| Deepak2014 | India | CT | 28(28) | 58.4±6.8 | 12 | 2 | 3.65 | 876 | ①⑩ | N |
|  |  | CG | 28(28) | 59.4±6.7 |  |  |  |  |  |  |
| Emery1998 | USA | CT | 30(15) | 65.4±6.4 | 10 | 4 | 3 | 810 | ②⑦⑧ | N |
|  |  | CG | 24(10) | 67.4±5.9 |  |  |  |  |  |  |
|  |  | CG | 25(12) | 67.4±7.1 |  |  |  |  |  |  |
| Karapolat2007 | Turkey | CT | 27(22) | 65.1±9.4 | 8 | 3 | 7 | 1260 | ①⑦⑧⑨⑩ | Y |
|  |  | CG | 22(21) | 66.6±8.4 |  |  |  |  |  |  |
| Ko2011 | China | CT | 30(30) | 73.47±7.72 | 8 | 3 | 5.4 | 1944 | ①②⑧⑩ | Y |
|  |  | CG | 30(29) | 73.80±6.35 |  |  |  |  |  |  |
| Lahham2020 | Australia | CT | 29(17) | 68±9 | 8 | 5 | 6.1 | 1830 | ① | Y |
|  |  | CG | 29(17) | 67±10 |  |  |  |  |  |  |
| Man2004 | Britain | CT | 21(9) | 69.6±9.2 | 8 | 2 | 3.72 | 446.4 | ③⑩ | Y |
|  |  | CG | 21(8) | 70.7±9.3 |  |  |  |  |  |  |
| Nakamura2008 | Japan | CT | 10 | 69.0±8.7 | 12 | 3 | 3.5 | 630 | ①②⑥ | N |
|  |  | CG | 10 | 69.9±7.1 |  |  |  |  |  |  |
| Ringbaek2000 | Denmark | CT | 24(1) | 61.8±6.8 | 8 | 2 | 4.2 | 1008 | ①⑩ | N |
|  |  | CG | 21(6) | 64.6±7.7 |  |  |  |  |  |  |
| Troosters2000 | Belgium | CT | 37(31) | 60±9 | 24 | 3 | 3.8 | 1026 | ⑥ | N |
|  |  | CG | 33(30) | 63±7 |  |  |  |  |  |  |
| Tsai2017 | Australia | CT | 19(12) | 73±8 | 8 | 3 | 6.2 | 1116 | ①③ | N |
|  |  | CG | 17(6) | 75±9 |  |  |  |  |  |  |
| Vasilopoulou2017 | Greece | CT | 47(44) | 66.9±9.6 | 48 | 3 | 5.9 | 1062 | ①⑩ | N |
|  |  | CT | 50(38) | 66.7±7.3 | 48 | 2 | 5.8 | 696 |  | N |
|  |  | CG | 50(37) | 64.0±8.0 |  |  |  |  |  |  |
| Wadell2013 | Canada | CT | 20(11) | 68±6 | 8 | 3 | 5.4 | 2430 | ①③⑩ | N |
|  |  | CG | 28(16) | 66±7 |  |  |  |  |  |  |
| Zambom-Ferraresi2015 | Spain | CT | 14(14) | 68±7 | 12 | 2 | 5.25 | 630 | ④⑤ | N |
|  |  | CG | 8(8) | 69±5 |  |  |  |  |  |  |

Note:①6-minute walk distance,6MWD;②Maximal oxygen uptake,VO_2max_;③Endurance shuttle walk test,ESWT;④Leg Press 1RM (LP);⑤Chest Press 1RM (CP);⑥Peak work rate (Wpeak);⑦Forced Vital Capacity (FVC);⑧Forced Expiratory Volume in one second (FEV_1_);⑨FEV_1_/FVC;⑩St.George's respiratory questionnaire,SGRQ；CT group (intervention): concurrent training;CG group (control): non-exercise intervention, usual care, wait-list control, or routine daily activities.

# 3.Risk of bias


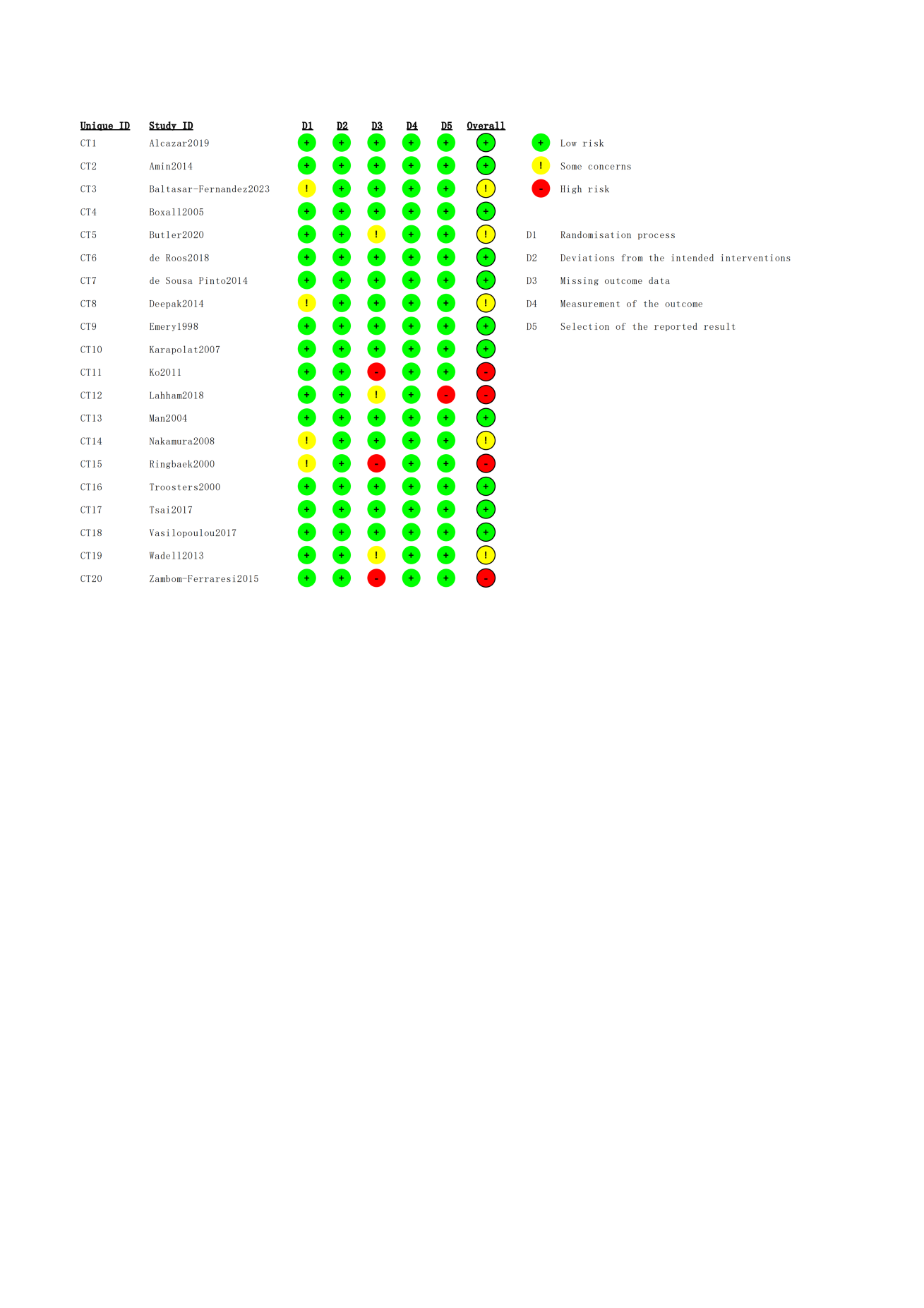


**
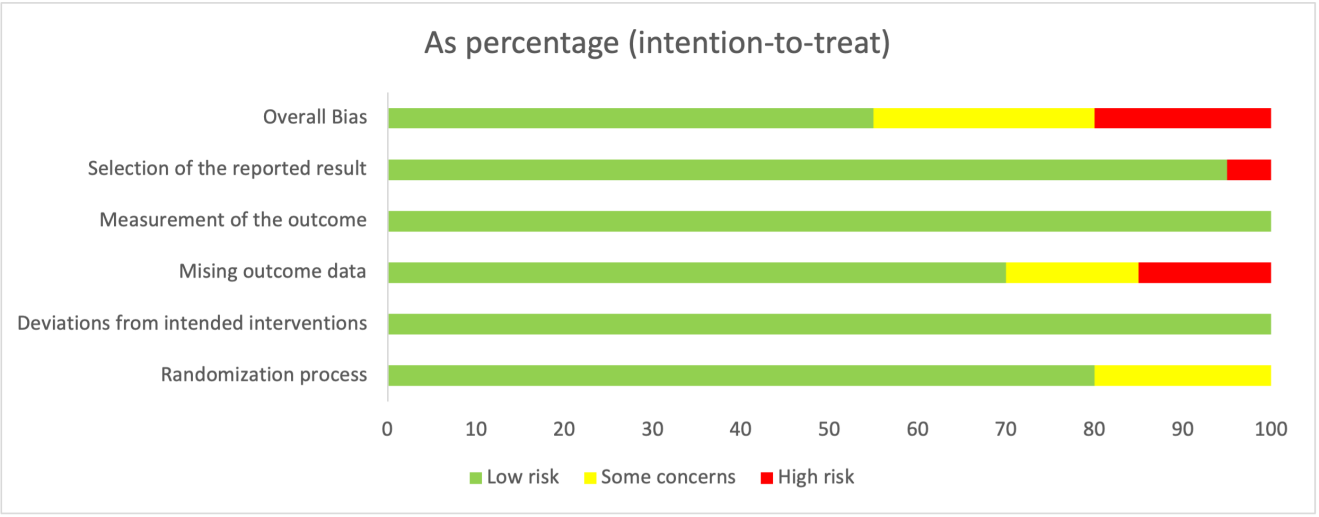
**

**Figure S3. Risk of bias**

# 4.Publication bias assessments


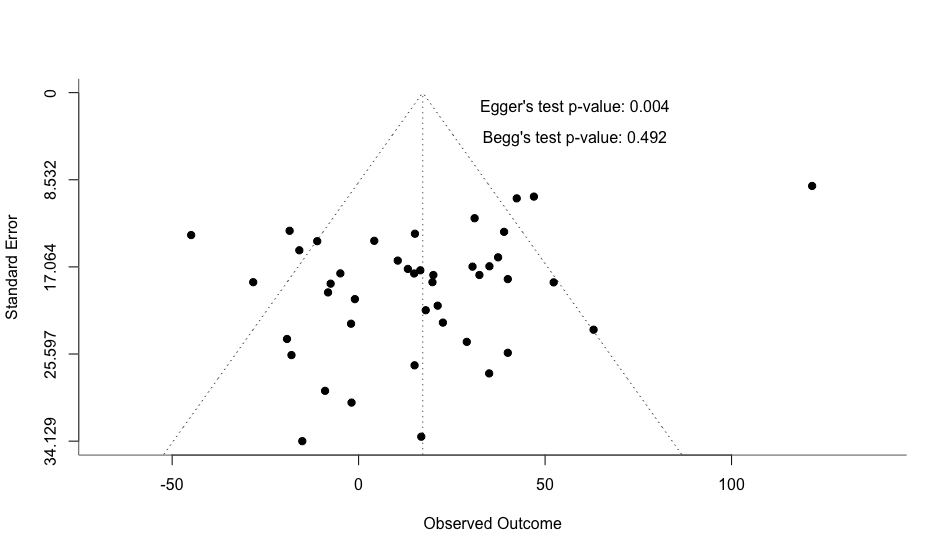


**Figure S4. Funnel plot of 6MWD**


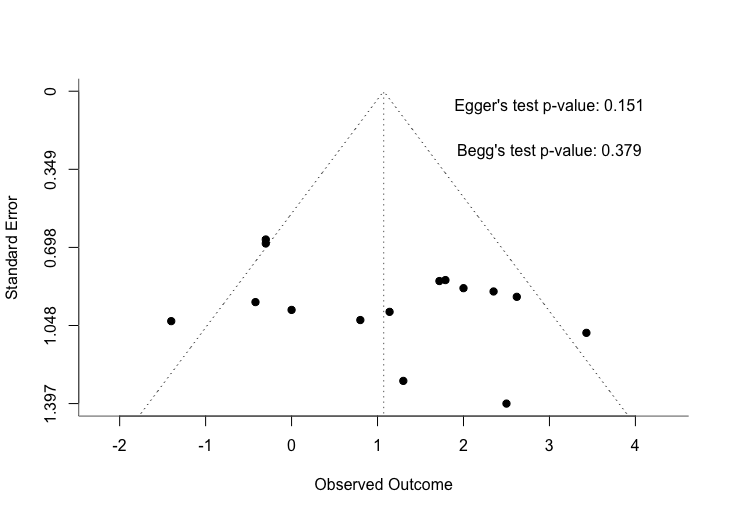


**Figure S4. Funnel plot of VO_2max_**


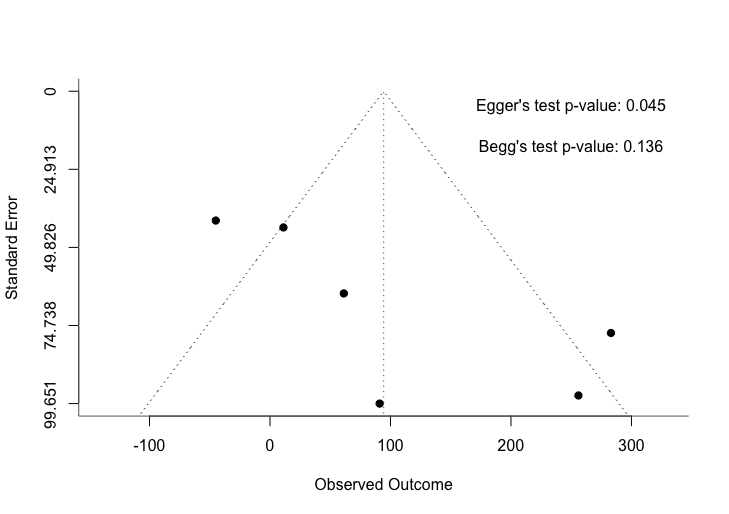


**Figure S4. Funnel plot of ESWT**


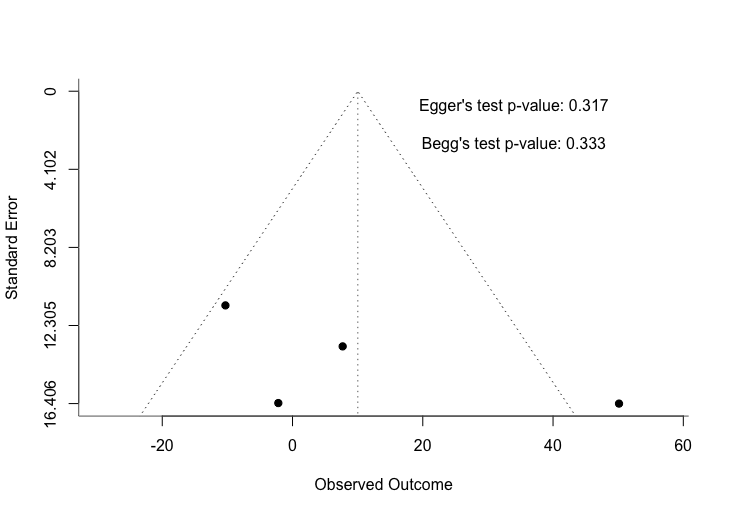


**Figure S4. Funnel plot of Leg Press 1RM**


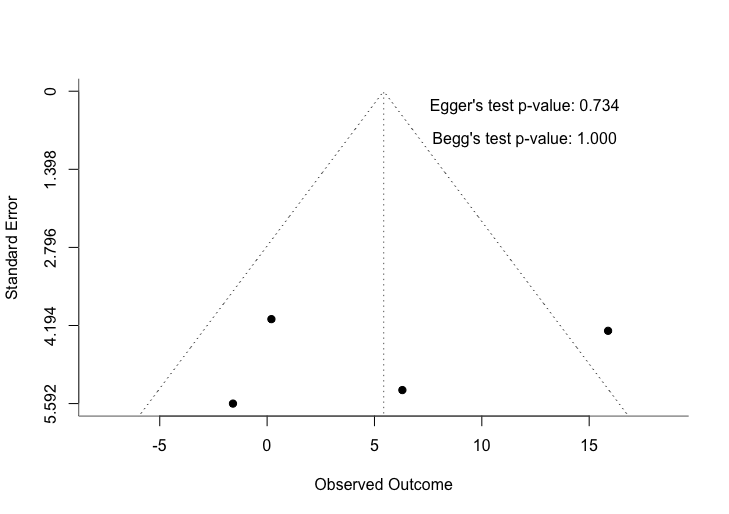


**Figure S4. Funnel plot of Chest Press 1RM**


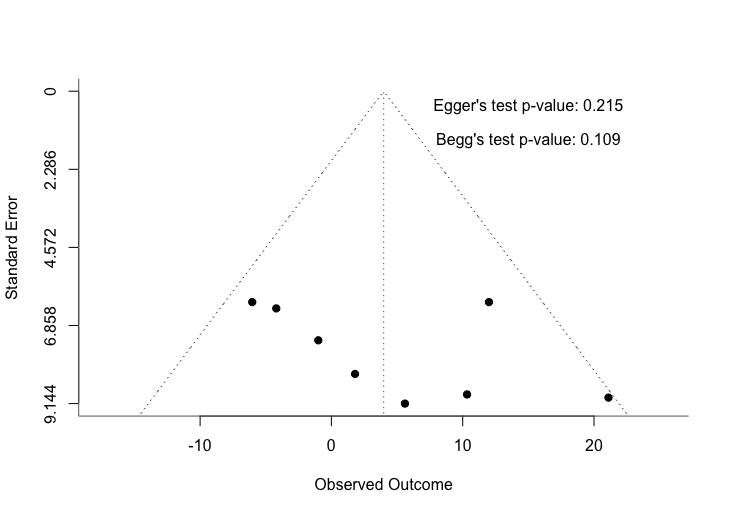


**Figure S4. Funnel plot of Wpeak**


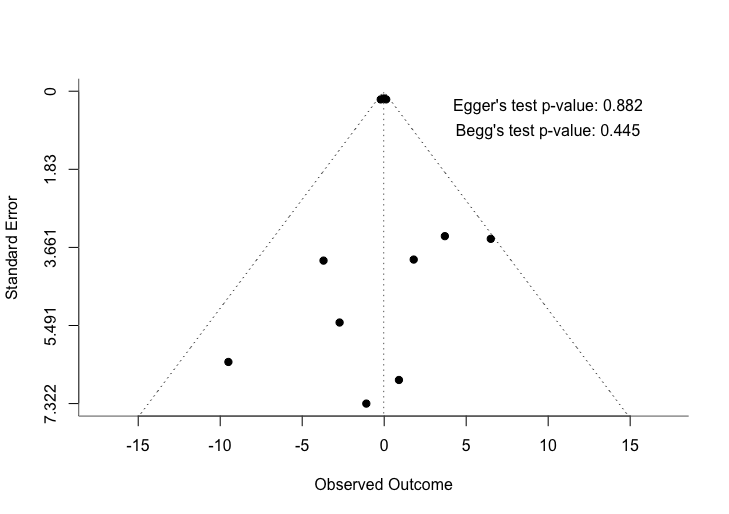


**Figure S4. Funnel plot of FVC**


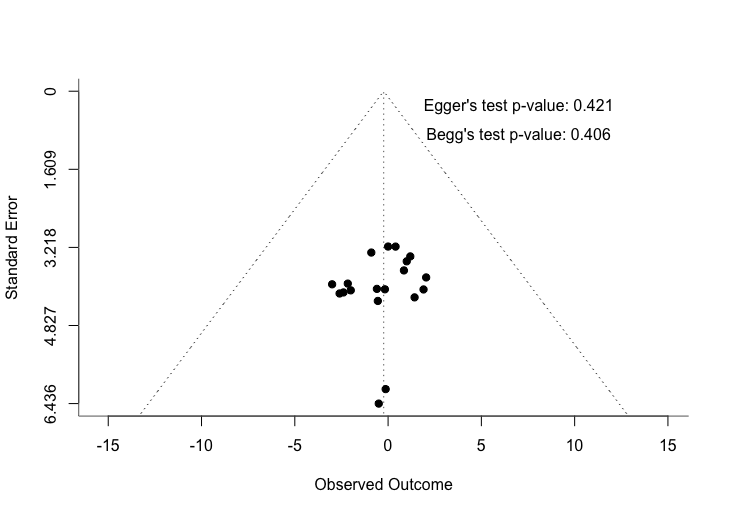


**Figure S4. Funnel plot of FEV_1_**

_
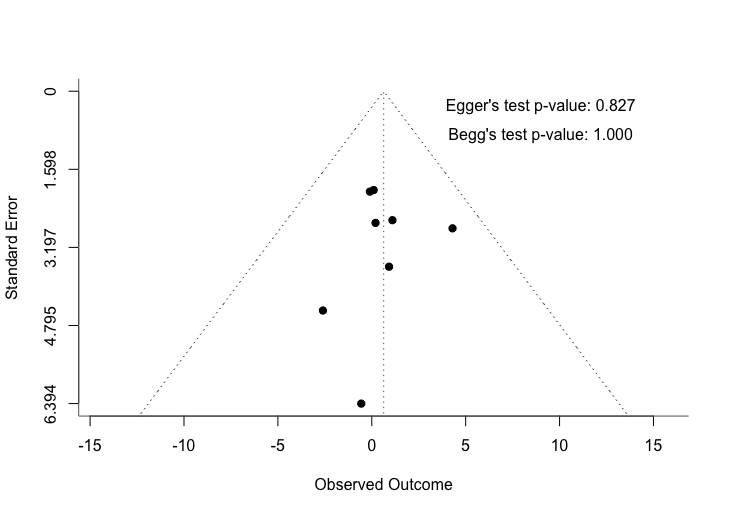
_

**Figure S4. Funnel plot of FEV_1_/FVC**

_
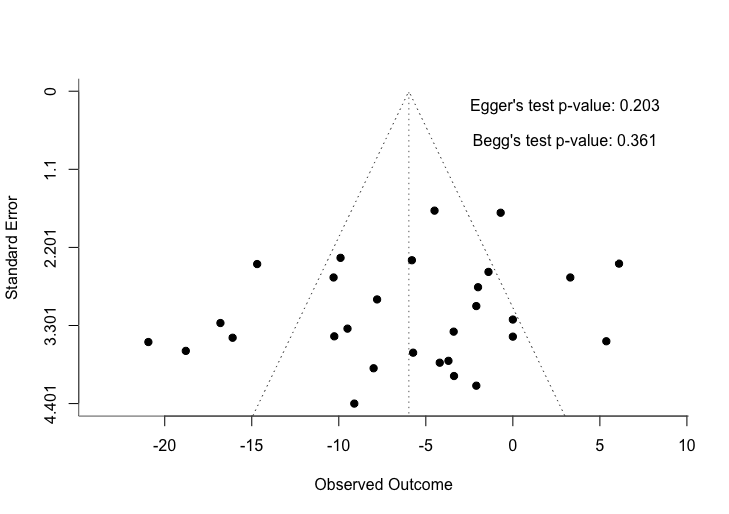
_

**Figure S4. Funnel plot of SGRQ**

# 5.Convergence and posterior distributions


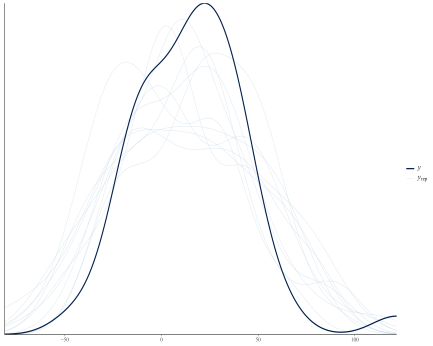

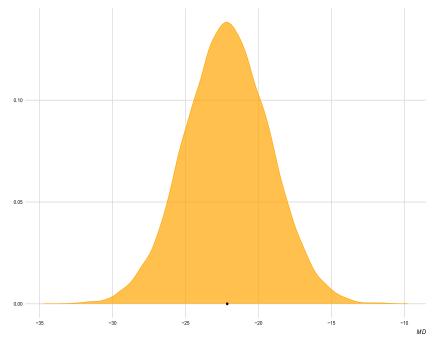

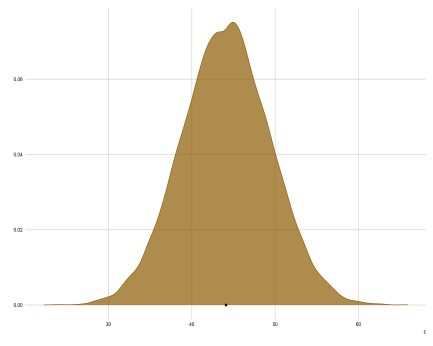


**Figure S5. Convergence and posterior distributions (6MWD).**


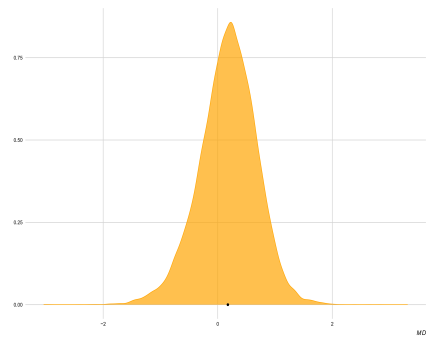

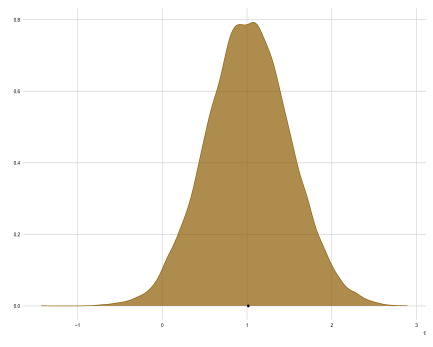

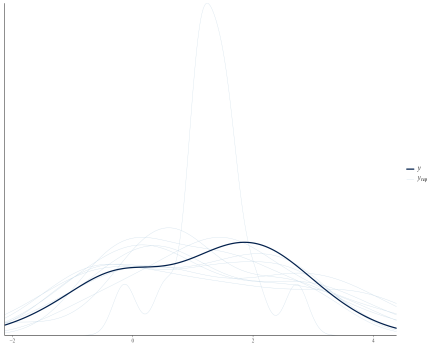


**Figure S5. Convergence and posterior distributions (VO_2max_).**


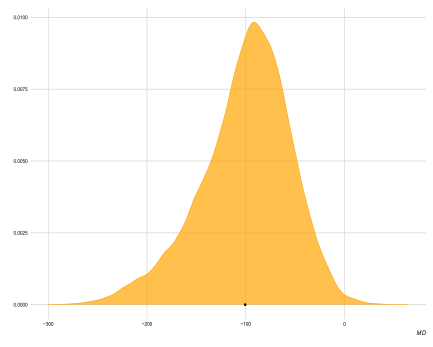

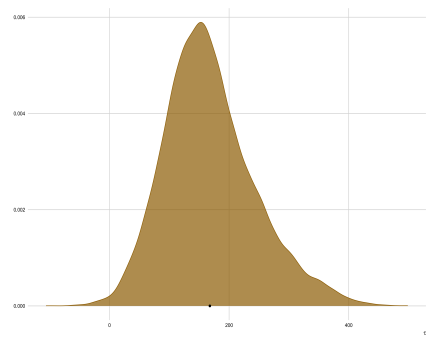

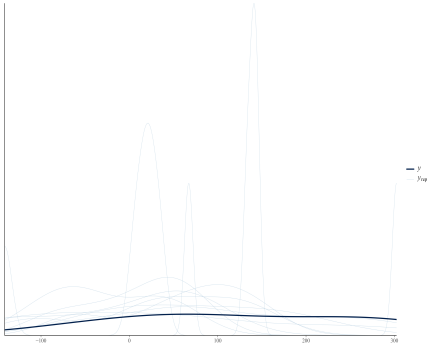


**Figure S5. Convergence and posterior distributions (ESWT).**


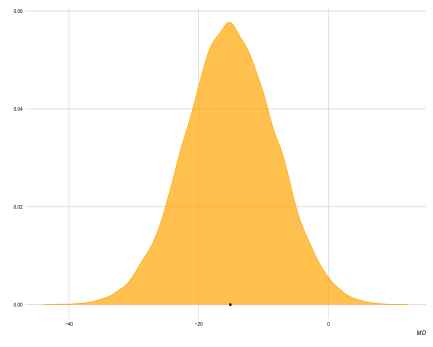

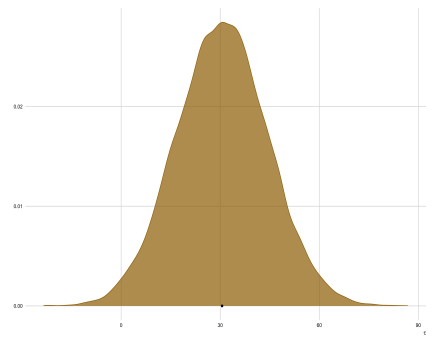

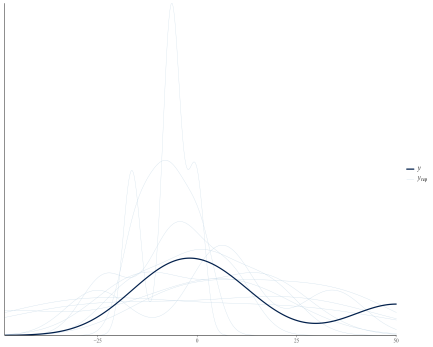


**Figure S5. Convergence and posterior distributions (LP 1RM).**


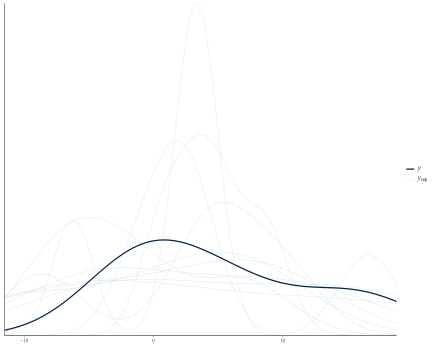

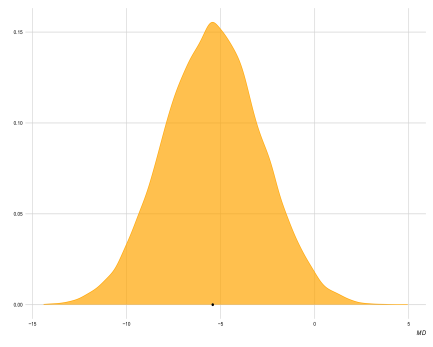

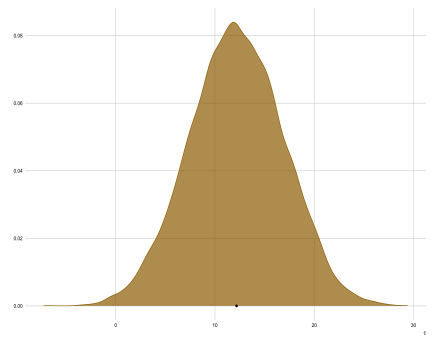


**Figure S5. Convergence and posterior distributions (CP 1RM).**


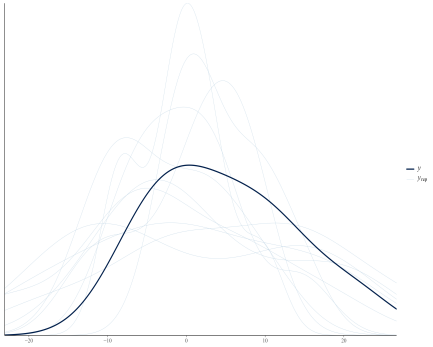

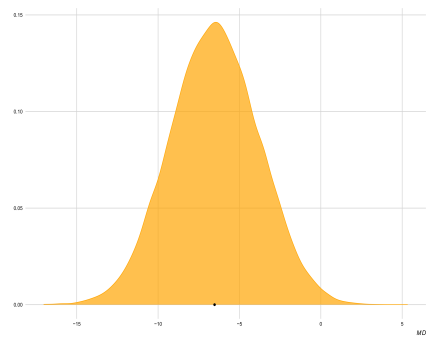

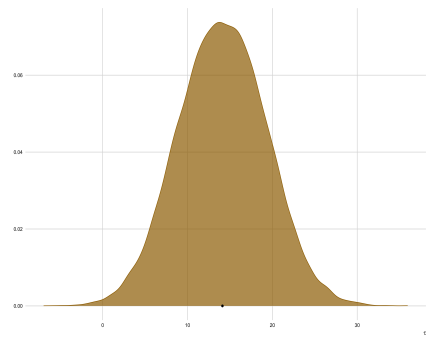


**Figure S5. Convergence and posterior distributions (Wpeak).**


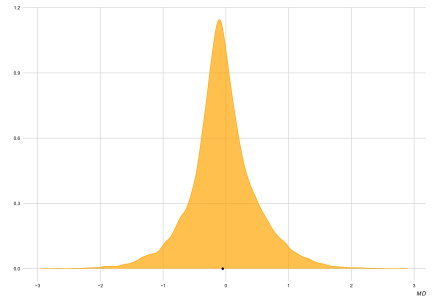

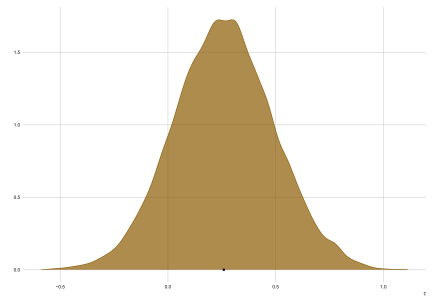

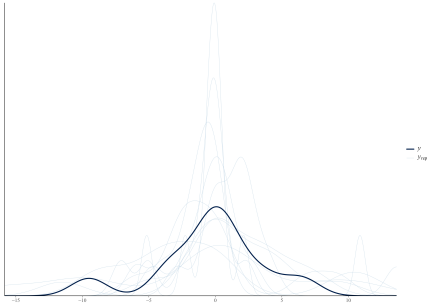


**Figure S5. Convergence and posterior distributions (FVC).**


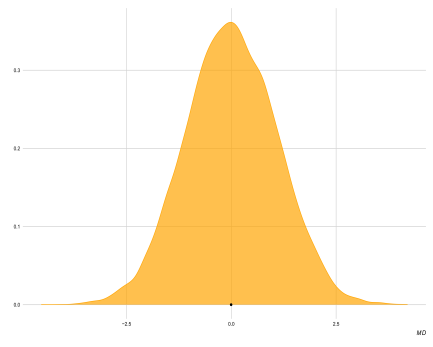

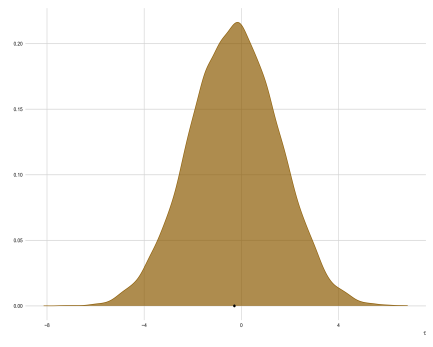

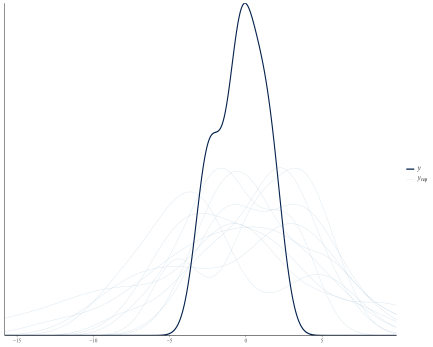


**Figure S5. Convergence and posterior distributions (FEV_1_).**


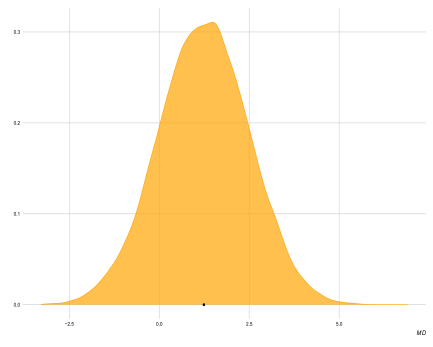

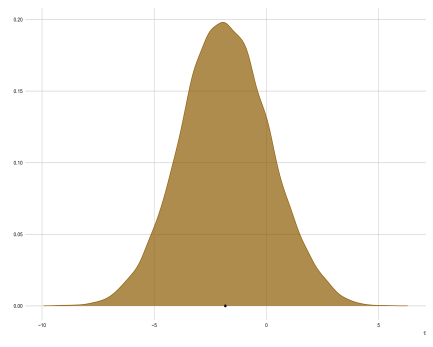

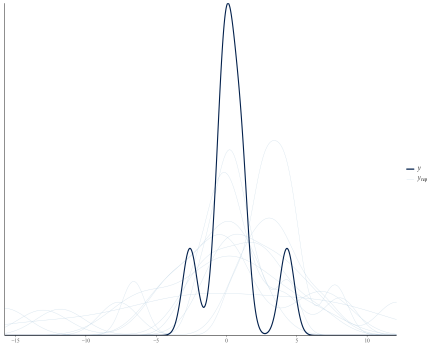


**Figure S5. Convergence and posterior distributions (FEV_1_/FVC).**


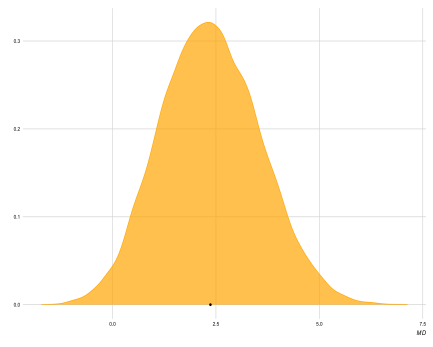

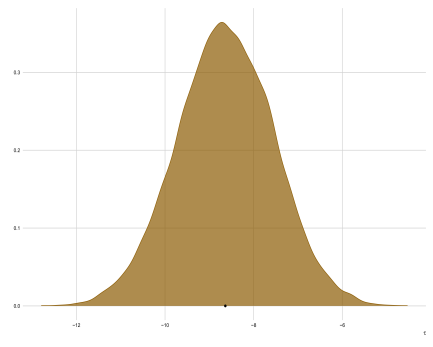

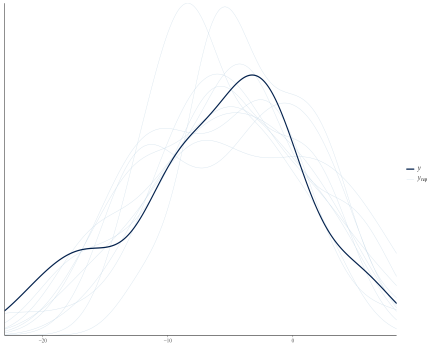


**Figure S5. Convergence and posterior distributions (SGRQ).**

# 6.Predicted dose-responses

| **weekly_dose** | **pred** | **se** | **lower** | **upper** |
| --- | --- | --- | --- | --- |
| 0 | 9.15009 | 40.33934 | -57.20221 | 75.50239 |
| 20 | 9.47247 | 39.49919 | -55.49792 | 74.44286 |
| 40 | 9.79486 | 38.65997 | -53.79514 | 73.38485 |
| 60 | 10.11724 | 37.82174 | -52.09399 | 72.32847 |
| 80 | 10.43962 | 36.98456 | -50.39457 | 71.27381 |
| 100 | 10.76201 | 36.14851 | -48.697 | 70.22101 |
| 120 | 11.08439 | 35.31366 | -47.00142 | 69.1702 |
| 140 | 11.40677 | 34.48011 | -45.30797 | 68.12151 |
| 160 | 11.72916 | 33.64795 | -43.6168 | 67.07511 |
| 180 | 12.05154 | 32.81729 | -41.92809 | 66.03117 |
| 200 | 12.37392 | 31.98824 | -40.24204 | 64.98989 |
| 220 | 12.69631 | 31.16093 | -38.55885 | 63.95147 |
| 240 | 13.01869 | 30.3355 | -36.87877 | 62.91615 |
| 260 | 13.34107 | 29.51212 | -35.20204 | 61.88418 |
| 280 | 13.66346 | 28.69095 | -33.52896 | 60.85587 |
| 300 | 13.98584 | 27.8722 | -31.85984 | 59.83153 |
| 320 | 14.30822 | 27.05608 | -30.19507 | 58.81151 |
| 340 | 14.63061 | 26.24284 | -28.53502 | 57.79624 |
| 360 | 14.95299 | 25.43275 | -26.88017 | 56.78615 |
| 380 | 15.27537 | 24.62614 | -25.23101 | 55.78176 |
| 400 | 15.59776 | 23.82334 | -23.58814 | 54.78366 |
| 420 | 15.92014 | 23.02475 | -21.95221 | 53.79249 |
| 440 | 16.24252 | 22.23084 | -20.32396 | 52.80901 |
| 460 | 16.56491 | 21.44212 | -18.70425 | 51.83406 |
| 480 | 16.88729 | 20.65919 | -17.09406 | 50.86864 |
| 500 | 17.20968 | 19.88273 | -15.4945 | 49.91385 |
| 520 | 17.53206 | 19.11352 | -13.90689 | 48.97101 |
| 540 | 17.85444 | 18.35249 | -12.33272 | 48.0416 |
| 560 | 18.17683 | 17.60069 | -10.77373 | 47.12738 |
| 580 | 18.49921 | 16.85935 | -9.23195 | 46.23037 |
| 600 | 18.82159 | 16.12992 | -7.70977 | 45.35295 |
| 620 | 19.14385 | 15.41419 | -6.21024 | 44.49793 |
| 640 | 19.46549 | 14.71443 | -4.73759 | 43.66858 |
| 660 | 19.78574 | 14.03323 | -3.29688 | 42.86836 |
| 680 | 20.10371 | 13.3732 | -1.89324 | 42.10067 |
| 700 | 20.41854 | 12.73685 | -0.53171 | 41.36879 |
| **720** | **20.72928** | **12.12658** | **0.78283** | **40.67573** |
| **740** | **21.03497** | **11.54458** | **2.04583** | **40.02411** |
| **760** | **21.33465** | **10.99271** | **3.25326** | **39.41605** |
| **780** | **21.62737** | **10.47243** | **4.40175** | **38.85298** |
| **800** | **21.91215** | **9.98473** | **5.48873** | **38.33556** |
| **820** | **22.18809** | **9.53005** | **6.51255** | **37.86362** |
| **840** | **22.45433** | **9.10831** | **7.4725** | **37.43616** |
| **860** | **22.71001** | **8.71881** | **8.36885** | **37.05118** |
| **880** | **22.9543** | **8.36024** | **9.20293** | **36.70567** |
| **900** | **23.18635** | **8.03065** | **9.9771** | **36.3956** |
| **920** | **23.40546** | **7.72783** | **10.6943** | **36.11661** |
| **940** | **23.61127** | **7.44989** | **11.3573** | **35.86524** |
| **960** | **23.8036** | **7.19525** | **11.96847** | **35.63873** |
| **980** | **23.98239** | **6.9628** | **12.5296** | **35.43518** |
| **1000** | **24.14769** | **6.75192** | **13.04176** | **35.25361** |
| **1020** | **24.29954** | **6.56229** | **13.50553** | **35.09354** |
| **1040** | **24.438** | **6.39387** | **13.92102** | **34.95498** |
| **1060** | **24.56311** | **6.24691** | **14.28786** | **34.83837** |
| **1080** | **24.67493** | **6.12189** | **14.60532** | **34.74454** |
| **1100** | **24.77351** | **6.01948** | **14.87234** | **34.67467** |
| **1120** | **24.85889** | **5.9405** | **15.08763** | **34.63014** |
| **1140** | **24.93112** | **5.88584** | **15.24976** | **34.61247** |
| **1160** | **24.99025** | **5.85642** | **15.35729** | **34.6232** |
| **1180** | **25.03633** | **5.85308** | **15.40887** | **34.66379** |
| **1200** | **25.06947** | **5.87668** | **15.4032** | **34.73574** |
| **1220** | **25.09989** | **5.92815** | **15.33894** | **34.84083** |
| **1240** | **25.09792** | **6.00845** | **15.21489** | **34.98094** |
| **1260** | **25.09396** | **6.11831** | **15.03023** | **35.15769** |
| **1280** | **25.07841** | **6.2581** | **14.78476** | **35.37206** |
| **1300** | **25.05165** | **6.42778** | **14.47889** | **35.62442** |
| **1320** | **25.0141** | **6.627** | **14.11365** | **35.91454** |
| **1340** | **24.96613** | **6.85506** | **13.69055** | **36.24171** |
| **1360** | **24.90814** | **7.11106** | **13.21149** | **36.6048** |
| **1380** | **24.84054** | **7.3939** | **12.67865** | **37.00243** |
| **1400** | **24.76371** | **7.70245** | **12.09431** | **37.43311** |
| **1420** | **24.67808** | **8.03567** | **11.46058** | **37.89558** |
| **1440** | **24.58407** | **8.39265** | **10.77939** | **38.38876** |
| **1460** | **24.48213** | **8.77252** | **10.05261** | **38.91165** |
| **1480** | **24.37268** | **9.17444** | **9.28207** | **39.46329** |
| **1500** | **24.25616** | **9.59763** | **8.46946** | **40.04286** |
| **1520** | **24.13299** | **10.04137** | **7.6164** | **40.64959** |
| **1540** | **24.00362** | **10.50502** | **6.7244** | **41.28285** |
| **1560** | **23.86848** | **10.98799** | **5.79483** | **41.94212** |
| **1580** | **23.72799** | **11.48978** | **4.82899** | **42.62699** |
| **1600** | **23.58257** | **12.00997** | **3.82792** | **43.33722** |
| **1620** | **23.43259** | **12.54832** | **2.79244** | **44.07274** |
| **1640** | **23.2784** | **13.10458** | **1.72328** | **44.83351** |
| **1660** | **23.12029** | **13.67854** | **0.62109** | **45.61949** |

# 7.Forest plot for pairwise comparison


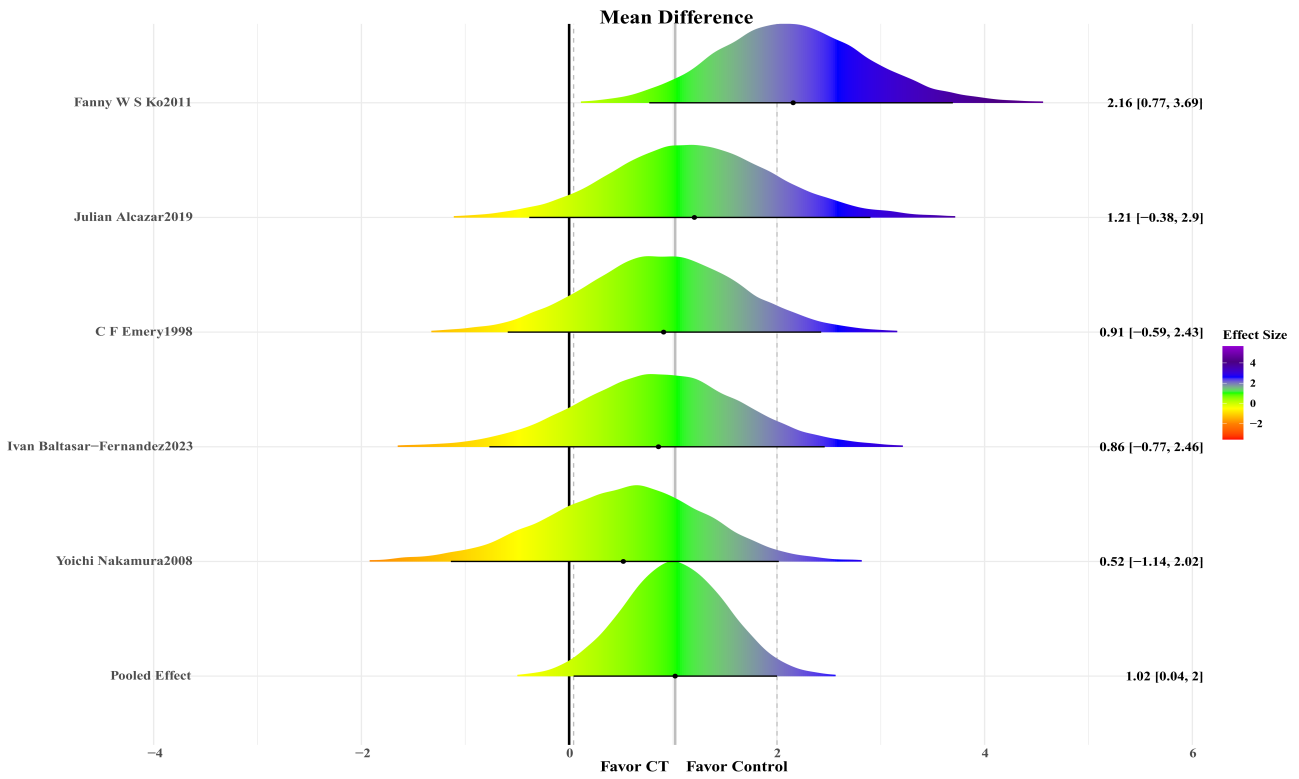


**Figure S7. Forest plot for pairwise comparison(VO_2max_).**

**
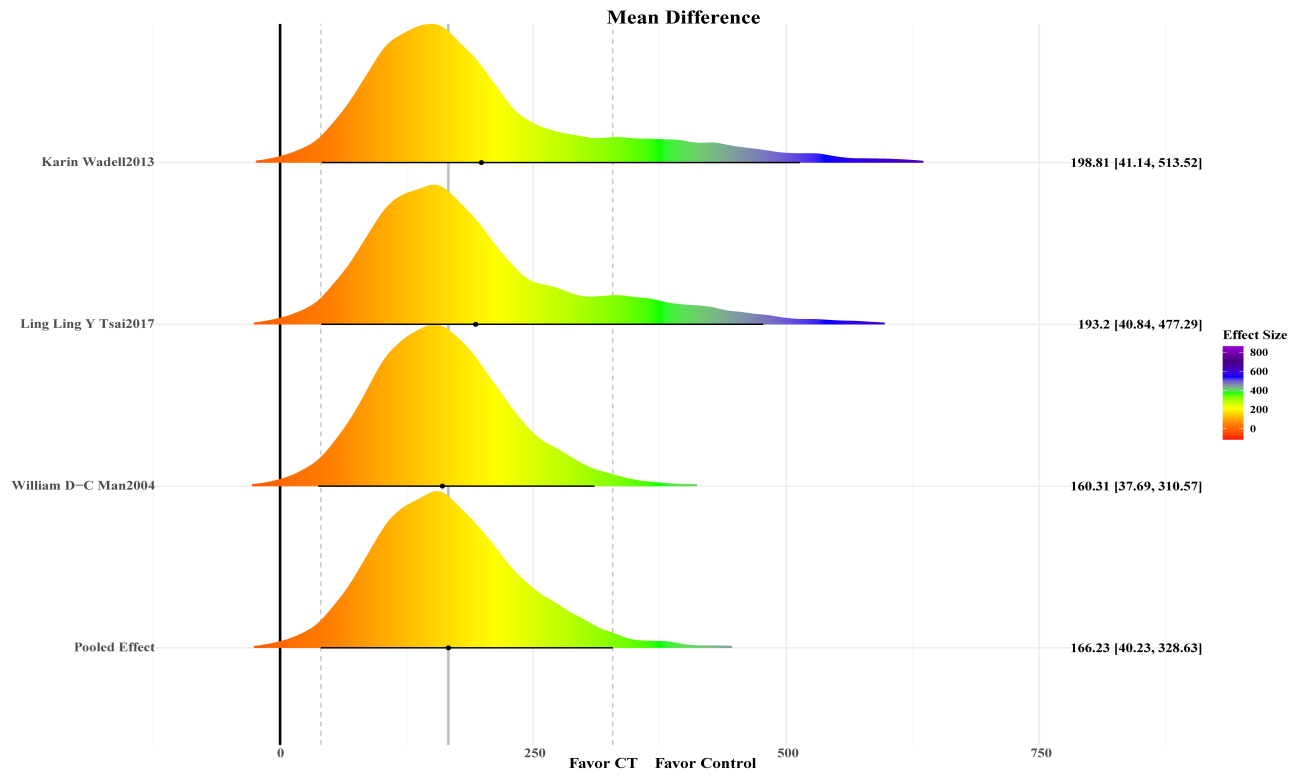
**

**Figure S7. Forest plot for pairwise comparison(ESWT).**

**
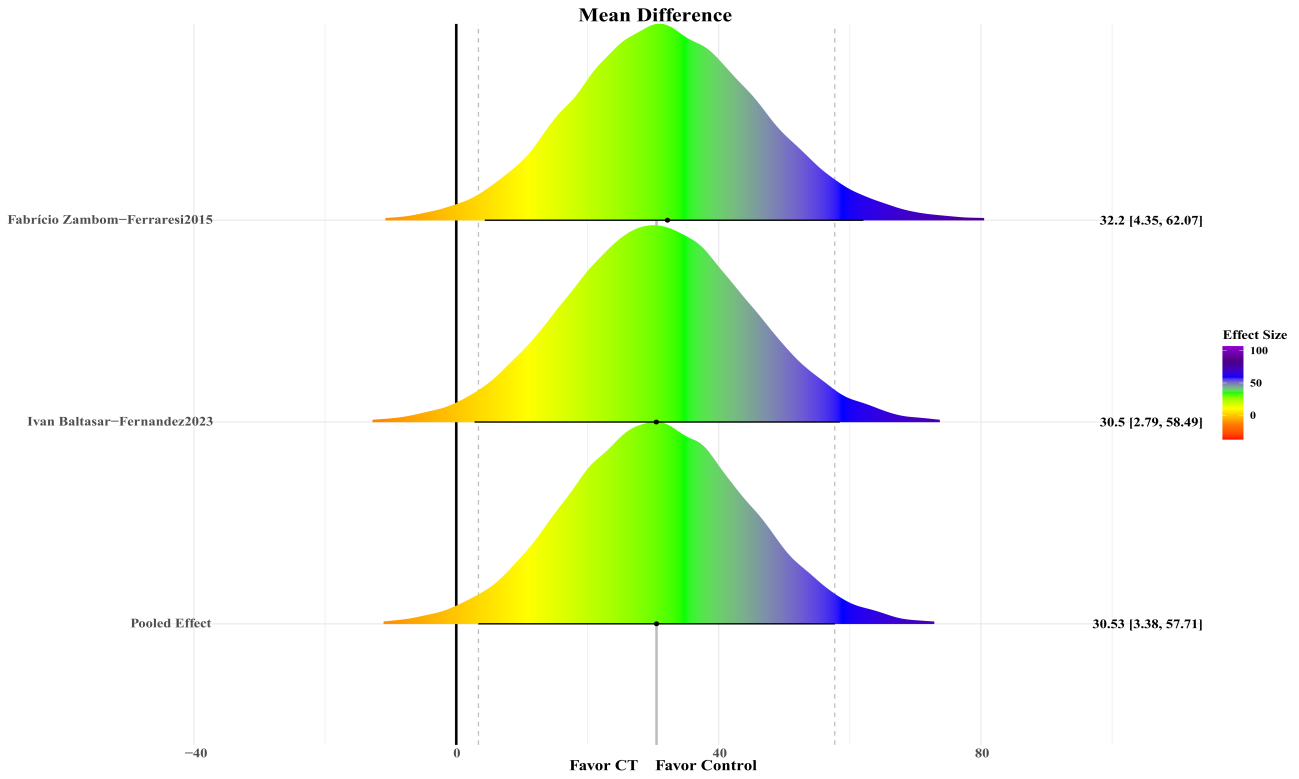
**

**Figure S7. Forest plot for pairwise comparison(Leg Press 1RM).**

**
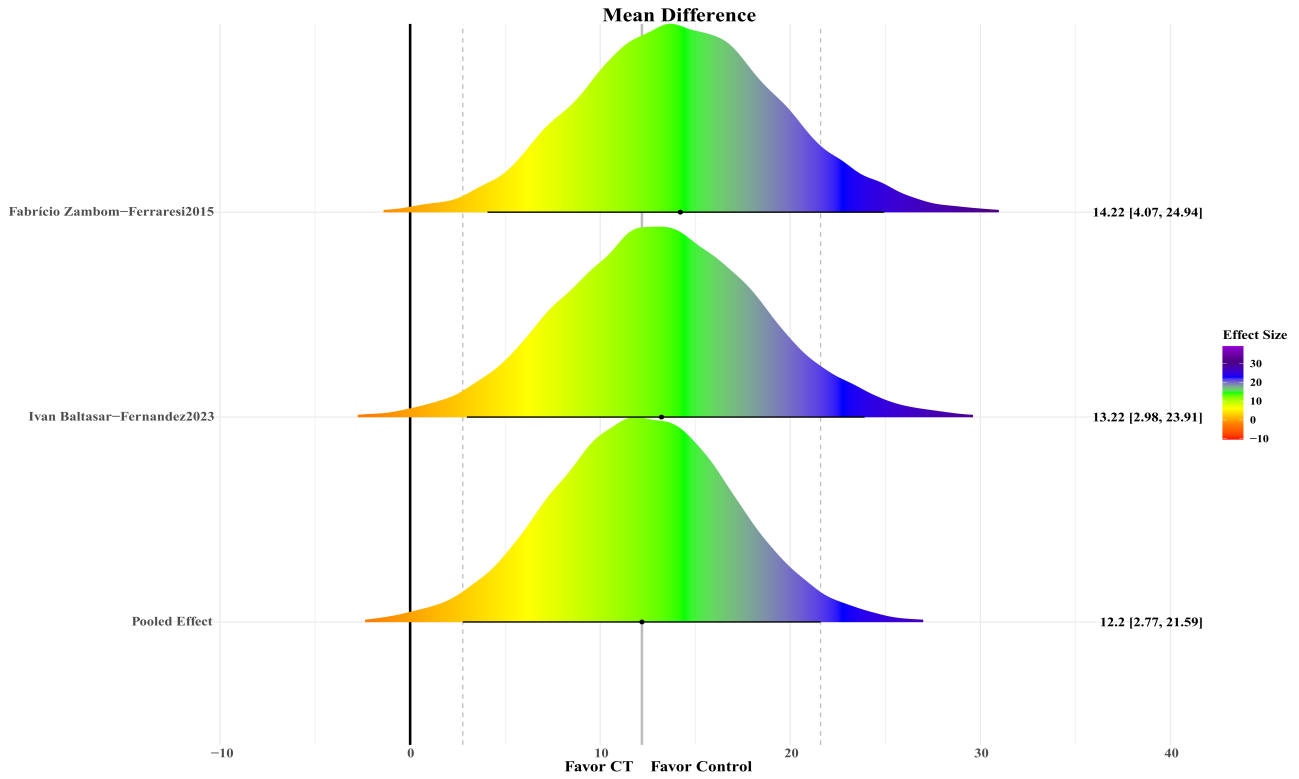
**

**Figure S7. Forest plot for pairwise comparison(Chest Press 1RM).**


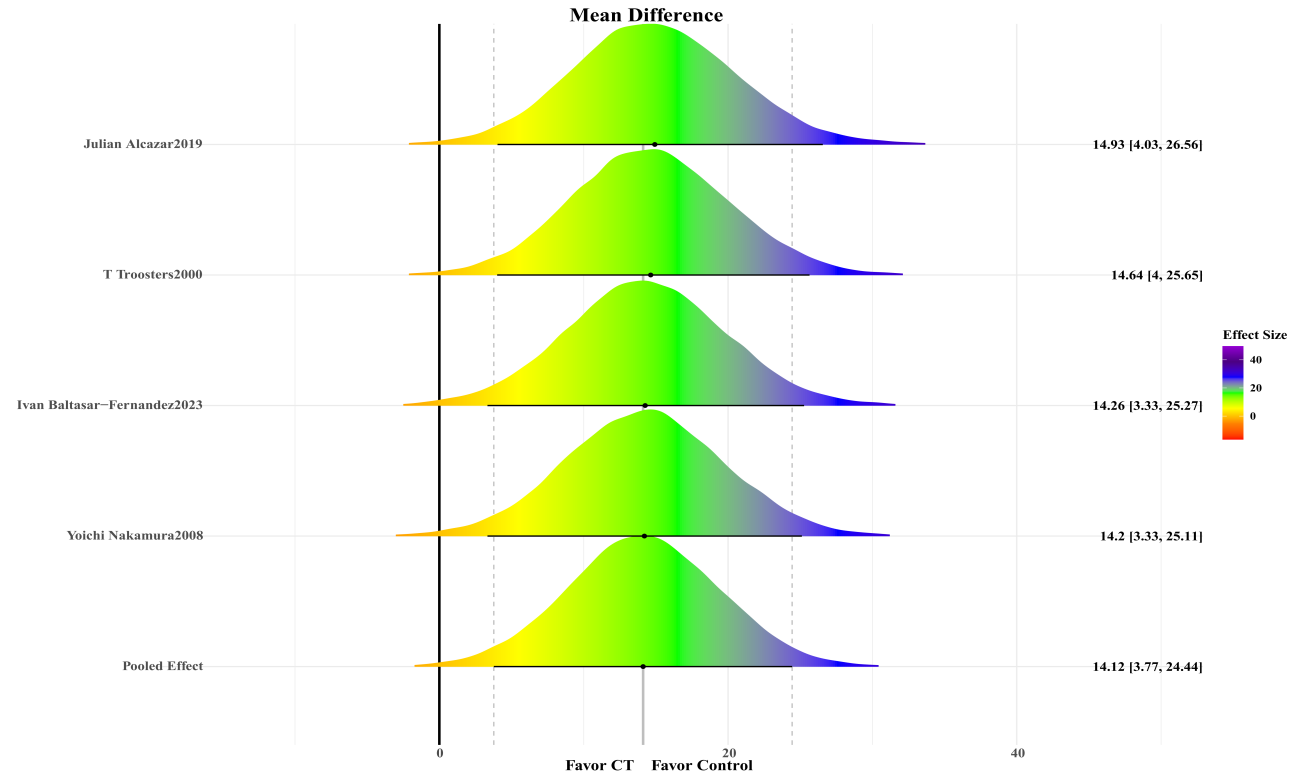


**Figure S7. Forest plot for pairwise comparison(Wpeak).**


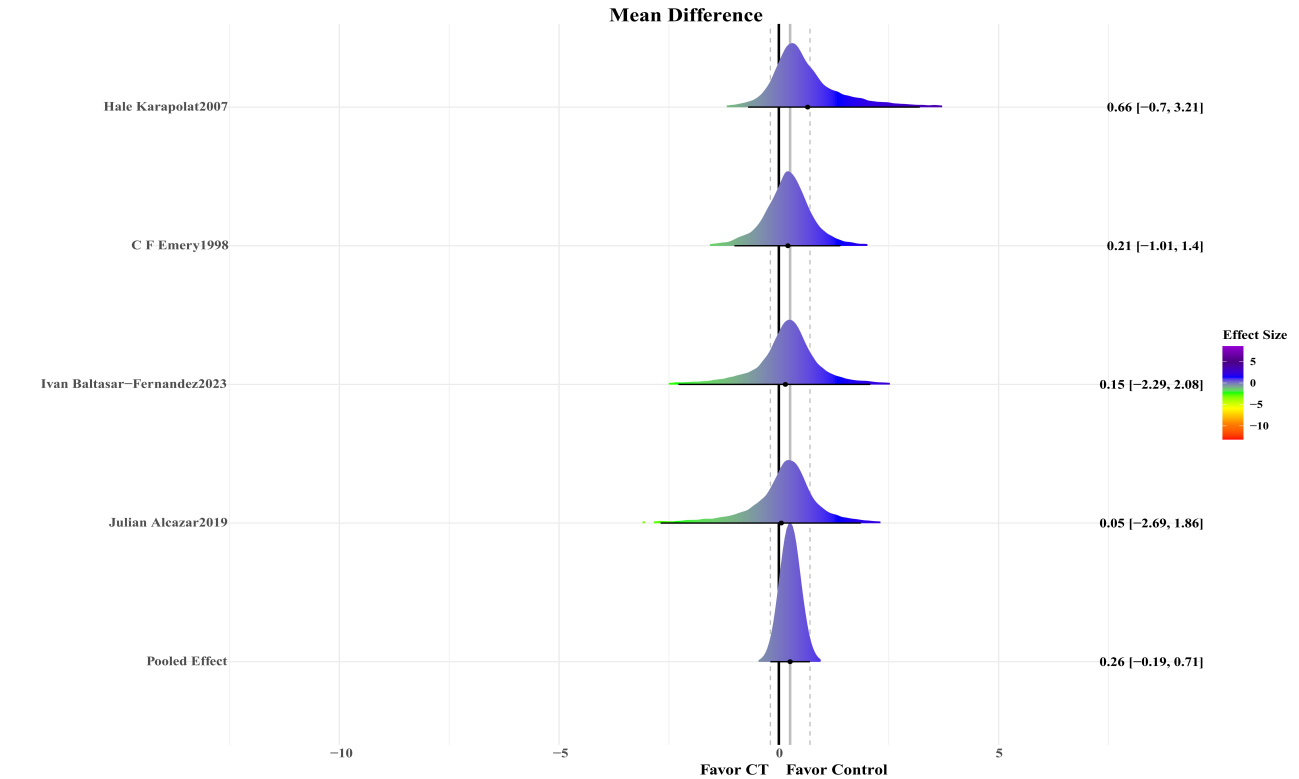


**Figure S7. Forest plot for pairwise comparison(FVC).**


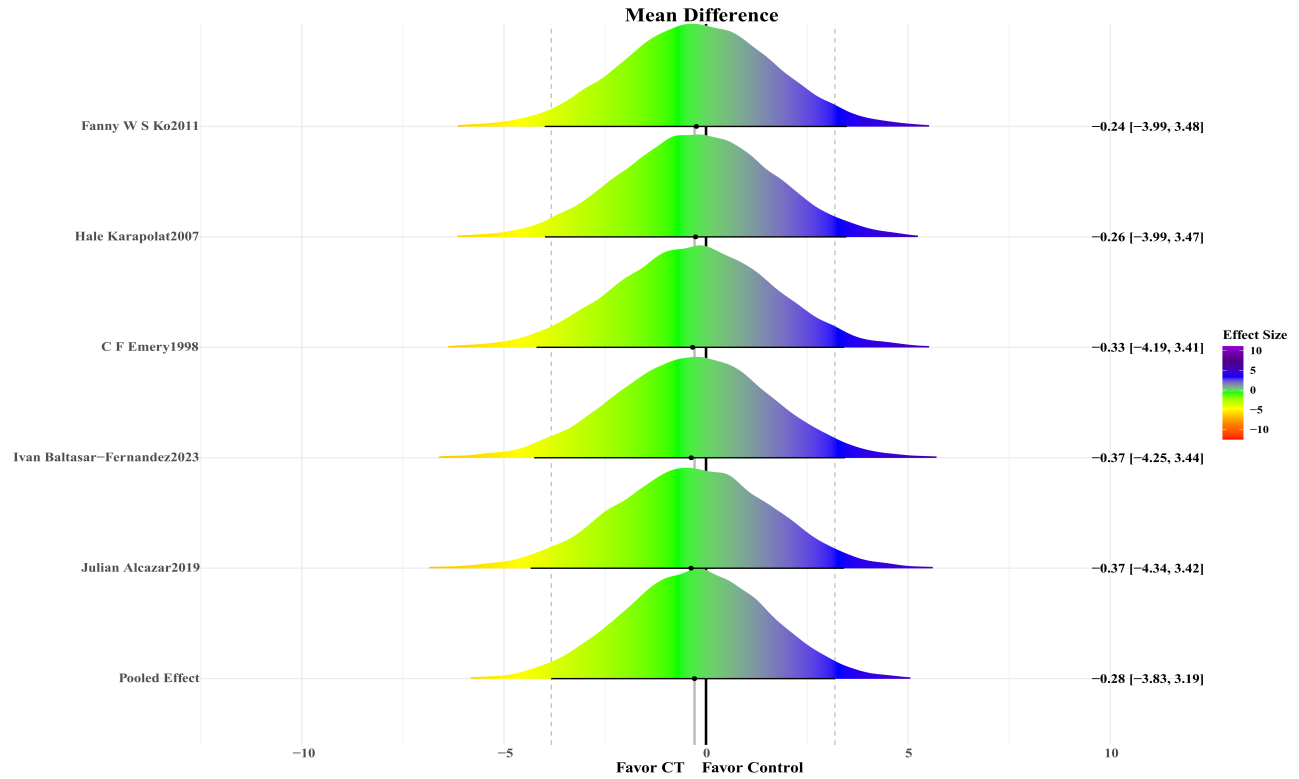


**Figure S7. Forest plot for pairwise comparison(FEV_1_).**


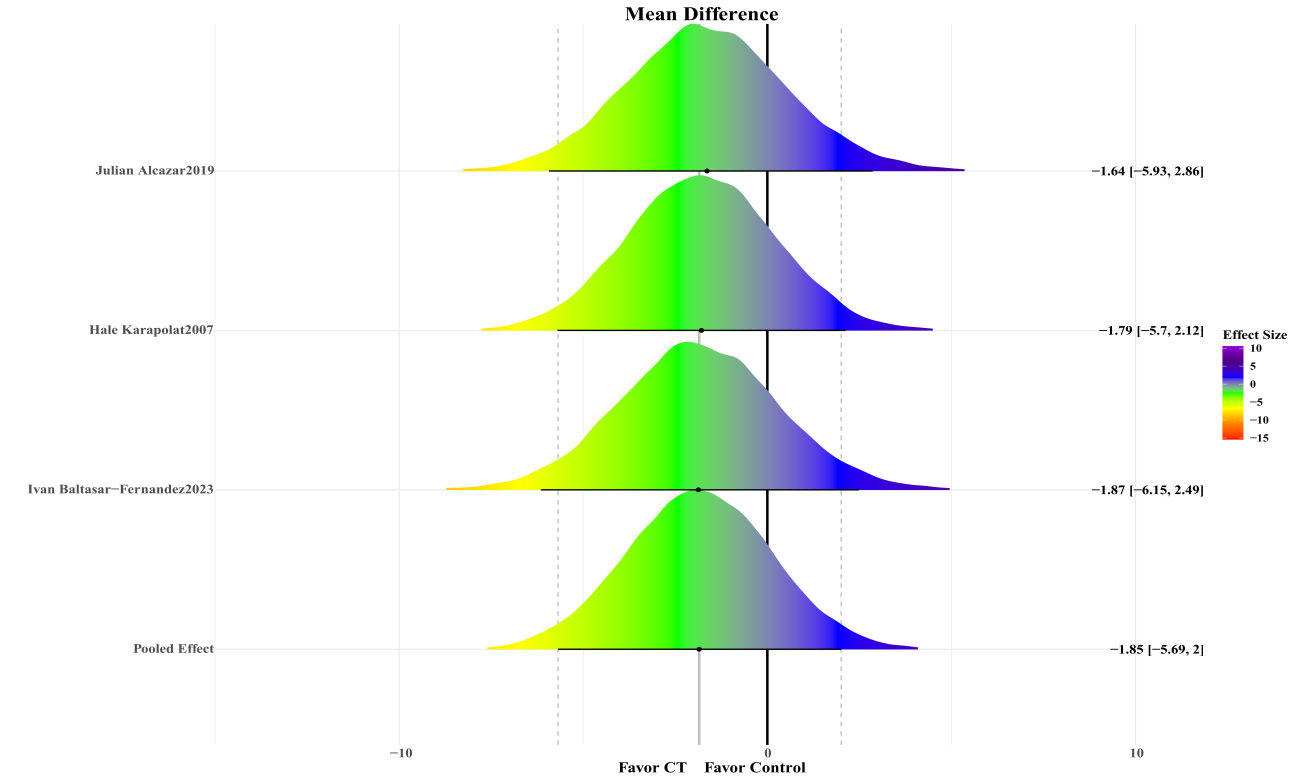


**Figure S7. Forest plot for pairwise comparison(FEV_1_/FVC).**

# 8.Meta‑regression


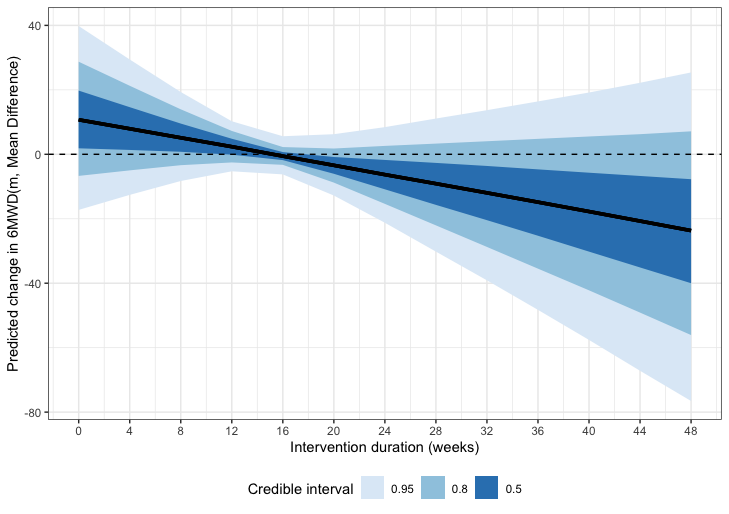


**Figure S8. Meta-regression analysis on intervention duration(6MWD).**


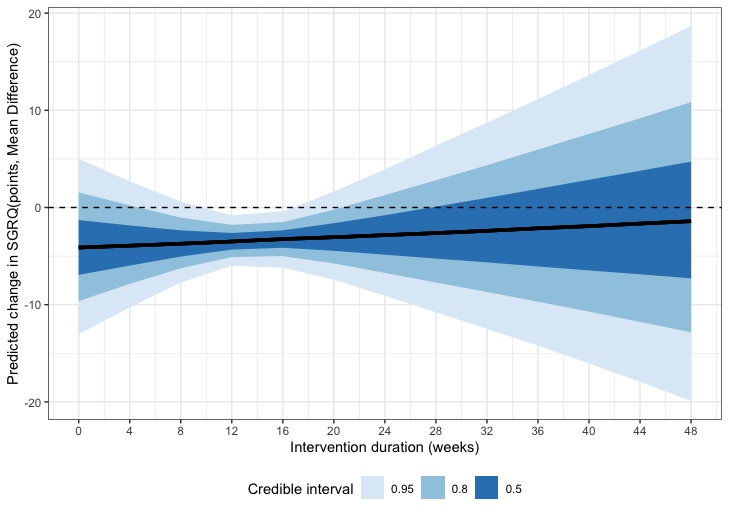


**Figure S8. Meta-regression analysis on intervention duration(SGRQ).**


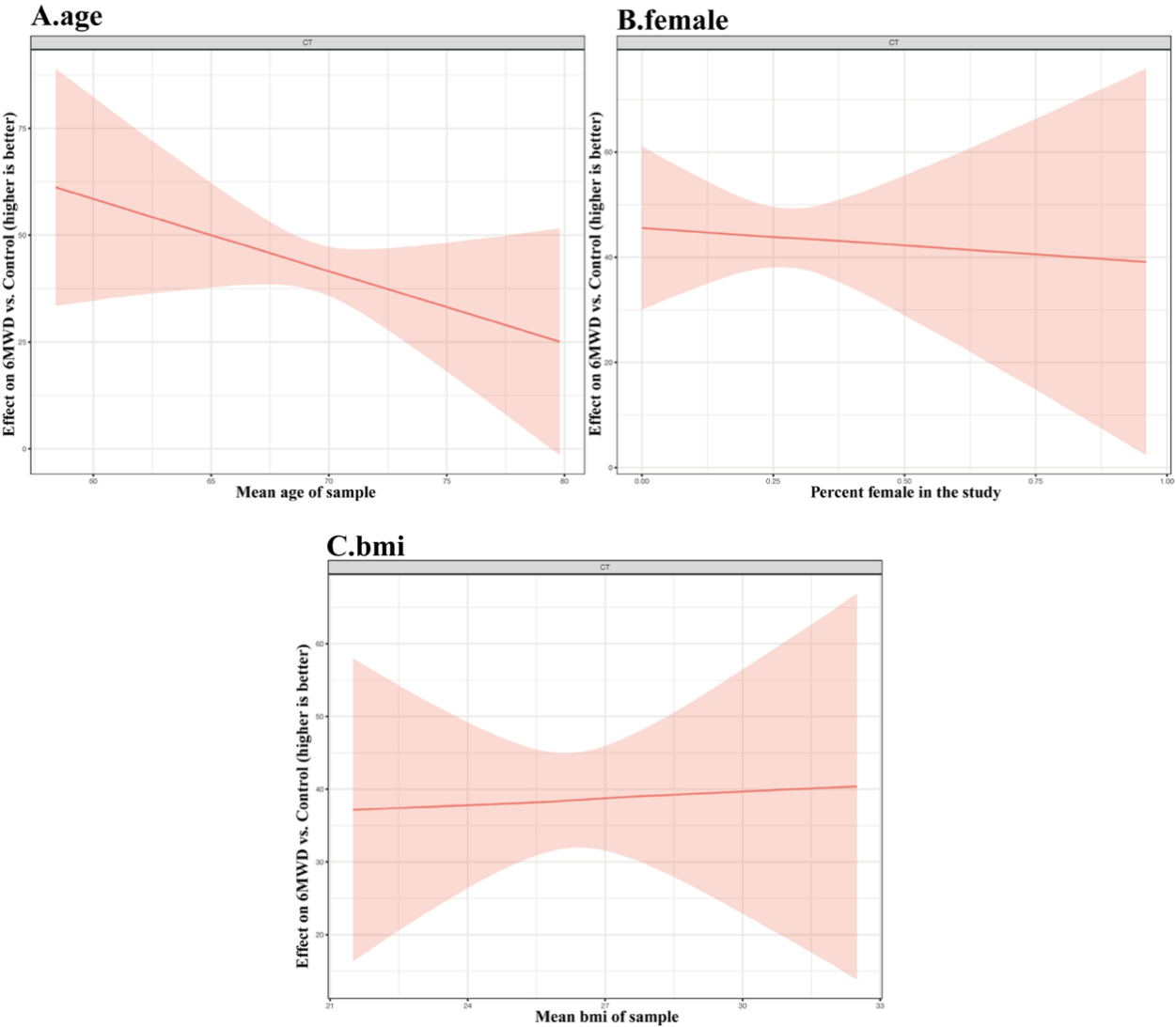


**Figure S8.Meta-regression analyses for six-minute walk distance (6MWD): A) Age; B) female; C) bmi**


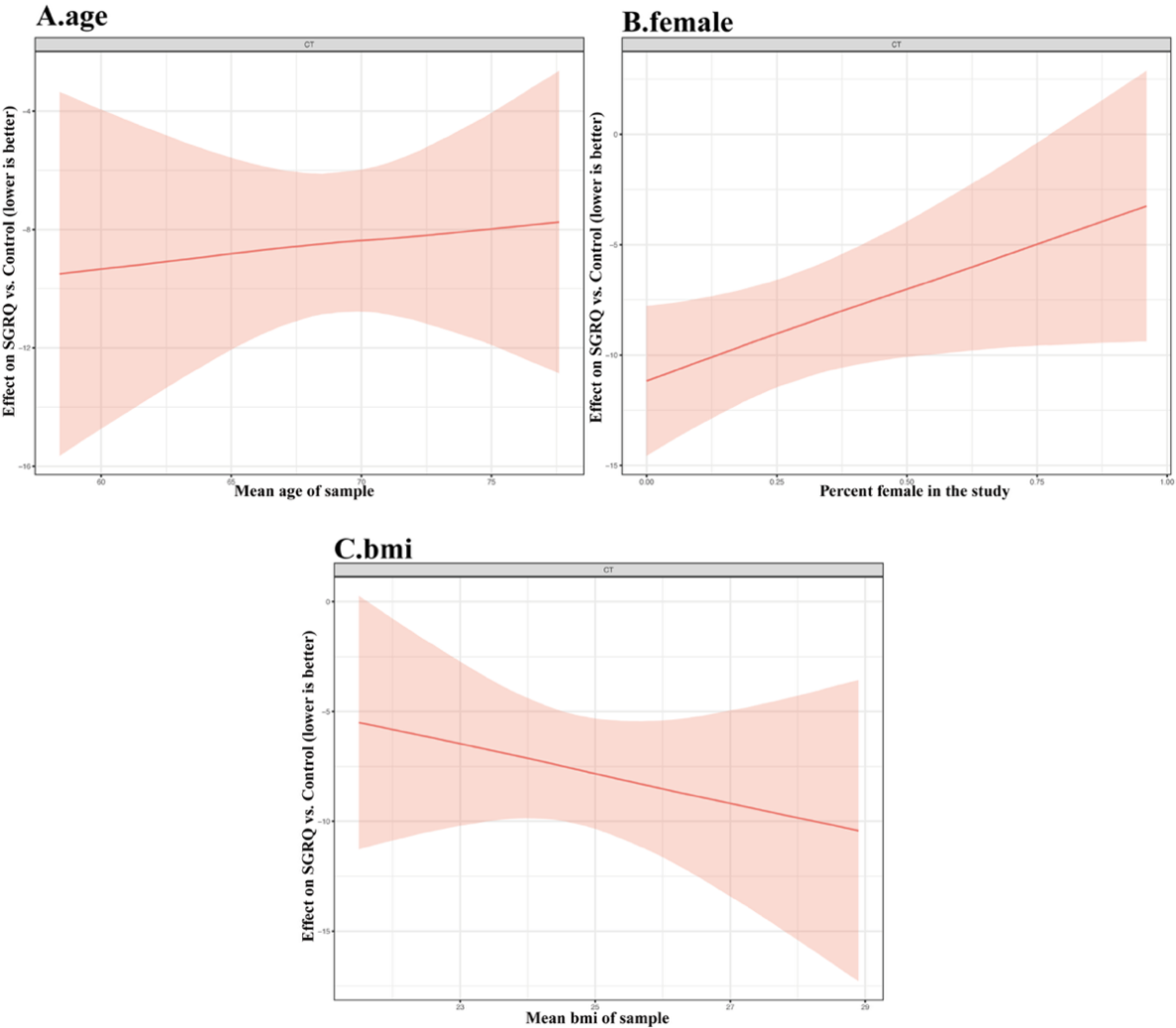


**Figure S8.Meta-regression analyses for SGRQ total: A) Age; B) female; C) bmi**

# 9.GRADE Evidence Quality Rating

GRADE Evidence Quality Rating for the Efficacy of Concurrent Training in Patients with Chronic Obstructive Pulmonary Disease (COPD)

| **Outcome** | **No. of Studies (Participants)** | **Certainty Assessment (Reasons for Downgrading/Upgrading)** | **Final GRADE Rating** |
| --- | --- | --- | --- |
| **Cardiopulmonary Fitness** | | | |
| 6MWD | 15 (755) | Downgraded for risk of bias¹ and inconsistency². Publication bias was detected⁵. | Very Low |
| VO_2max_ | 5 (184) | Downgraded for risk of bias¹, inconsistency⁸, and serious imprecision⁹. | Very Low |
| ESWT | 3 (126) | Downgraded for risk of bias¹, inconsistency¹⁰, and serious imprecision¹¹. | Very Low |
| **Muscle Strength** | | | |
| LP 1RM | 2 (43) | Downgraded for risk of bias¹, inconsistency¹², and very serious imprecision¹³. | Very Low |
| CP 1RM | 2 (43) | Downgraded for risk of bias¹, inconsistency¹⁴, and very serious imprecision¹³. | Very Low |
| Wpeak | 4 (140) | Downgraded for risk of bias¹, inconsistency¹⁵, and serious imprecision¹⁶. | Very Low |
| **Pulmonary Function** | | | |
| FVC | 4 (153) | Downgraded for risk of bias¹, inconsistency¹⁷, and very serious imprecision¹⁸. | Very Low |
| FEV_1_ | 5 (213) | Downgraded for risk of bias¹, inconsistency¹⁹, and very serious imprecision¹⁸. | Very Low |
| FEV_1_/FVC | 3 (99) | Downgraded for risk of bias¹, inconsistency²⁰, and very serious imprecision¹⁸. | Very Low |
| **Quality of Life** | | | |
| SGRQ | 10 (503) | Downgraded for risk of bias¹ and inconsistency⁶. Upgraded for a large, clinically significant effect⁷. | Moderate |

Explanations for Downgrading/Upgrading:

1. Risk of Bias: Downgraded due to methodological limitations across included studies. The author notes that "the overall quality of the included studies was relatively low, as some randomized controlled trials did not implement adequate blinding procedures". The risk of bias assessment figure also shows a mix of low risk, some concerns, and high risk studies.
2. Inconsistency (6MWD): Downgraded due to "high heterogeneity" reported in the analysis.
3. Large Effect (6MWD): Upgraded because the mean difference of 44.08 meters exceeds the established minimal clinically important difference (MCID) for COPD patients (typically 25-35 meters).
4. Dose-Response (6MWD): Upgraded because a clear dose-response relationship was identified, with an optimal dose of 1220 MET-min/week.
5. Publication Bias (6MWD): The analysis found evidence of publication bias for this outcome using Egger's and Begg's tests.
6. Inconsistency (SGRQ): Downgraded due to "high heterogeneity" reported in the analysis.
7. Large Effect (SGRQ): Upgraded because the mean difference of -8.65 points is more than double the MCID of -4 points for the SGRQ score.
8. Inconsistency (VO_2max_): Downgraded due to "high heterogeneity".
9. Imprecision (VO_2max_): The 95% credible interval (0.04 to 2.00) is very close to the line of no effect.
10. Inconsistency (ESWT): Downgraded due to "moderate heterogeneity".
11. Imprecision (ESWT): The 95% credible interval (40.23 to 328.63) is extremely wide.
12. Inconsistency (LP 1RM): Downgraded due to "high heterogeneity".
13. Very Serious Imprecision (LP & CP 1RM): The analyses were based on only two studies with a very small total sample size (N=43), and the credible intervals are very wide.
14. Inconsistency (CP 1RM): Downgraded due to "moderate heterogeneity".
15. Inconsistency (Wpeak): Downgraded due to "moderate heterogeneity".
16. Imprecision (Wpeak): The 95% credible interval (3.77 to 24.44) is wide for the effect size.
17. Inconsistency (FVC): Downgraded due to "high heterogeneity".
18. Very Serious Imprecision (Pulmonary Function): For FVC, FEV_1_, and FEV_1_/FVC, the 95% credible intervals all cross the line of no effect, indicating a non-significant finding.
19. Inconsistency (FEV_1_): Downgraded due to "moderate heterogeneity".
20. Inconsistency (FEV_1_/FVC): Downgraded due to "moderate heterogeneity".

# 10.Included studies

1. Alcazar J, Losa-Reyna J, Rodriguez-Lopez C, Navarro-Cruz R, Alfaro-Acha A, Ara I, García-García FJ, Alegre LM, Guadalupe-Grau A. Effects of concurrent exercise training on muscle dysfunction and systemic oxidative stress in older people with COPD. Scand J Med Sci Sports. 2019 Oct;29(10):1591-1603. doi: 10.1111/sms.13494. Epub 2019 Jun 24. PMID: 31169924.
2. Amin S, Abrazado M, Quinn M, Storer TW, Tseng CH, Cooper CB. A controlled study of community-based exercise training in patients with moderate COPD. BMC Pulm Med. 2014 Aug 4;14:125. doi: 10.1186/1471-2466-14-125. PMID: 25088030; PMCID: PMC4124480.
3. Baltasar-Fernandez I, Losa-Reyna J, Carretero A, Rodriguez-Lopez C, Alfaro-Acha A, Guadalupe-Grau A, Ara I, Alegre LM, Gomez-Cabrera MC, García-García FJ, Alcazar J. Residual effects of 12 weeks of power-oriented resistance training plus high-intensity interval training on muscle dysfunction, systemic oxidative damage, and antioxidant capacity after 10 months of training cessation in older people with COPD. Scand J Med Sci Sports. 2023 Sep;33(9):1661-1676. doi: 10.1111/sms.14428. Epub 2023 Jun 15. PMID: 37322570.
4. Boxall AM, Barclay L, Sayers A, Caplan GA. Managing chronic obstructive pulmonary disease in the community. A randomized controlled trial of home-based pulmonary rehabilitation for elderly housebound patients. J Cardiopulm Rehabil. 2005 Nov-Dec;25(6):378-85. doi: 10.1097/00008483-200511000-00012. PMID: 16327534.
5. Butler SJ, Desveaux L, Lee AL, Beauchamp MK, Brusco NK, Wang W, Goldstein RS, Brooks D. Randomized controlled trial of community-based, post-rehabilitation exercise in COPD. Respir Med. 2020 Nov-Dec;174:106195. doi: 10.1016/j.rmed.2020.106195. Epub 2020 Oct 15. PMID: 33171390.
6. de Roos P, Lucas C, Strijbos JH, van Trijffel E. Effectiveness of a combined exercise training and home-based walking programme on physical activity compared with standard medical care in moderate COPD: a randomised controlled trial. Physiotherapy. 2018 Mar;104(1):116-121. doi: 10.1016/j.physio.2016.08.005. Epub 2017 Jul 14. PMID: 28802772.
7. de Sousa Pinto JM, Martín-Nogueras AM, Calvo-Arenillas JI, Ramos-González J. Clinical benefits of home-based pulmonary rehabilitation in patients with chronic obstructive pulmonary disease. J Cardiopulm Rehabil Prev. 2014 Sep-Oct;34(5):355-9. doi: 10.1097/HCR.0000000000000061. PMID: 24866357.
8. Deepak TH, Mohapatra PR, Janmeja AK, Sood P, Gupta M. Outcome of pulmonary rehabilitation in patients after acute exacerbation of chronic obstructive pulmonary disease. Indian J Chest Dis Allied Sci. 2014 Jan-Mar;56(1):7-12. PMID: 24930201.
9. Emery CF, Schein RL, Hauck ER, MacIntyre NR. Psychological and cognitive outcomes of a randomized trial of exercise among patients with chronic obstructive pulmonary disease. Health Psychol. 1998 May;17(3):232-40. doi: 10.1037//0278-6133.17.3.232. PMID: 9619472.
10. Karapolat H, Atasever A, Atamaz F, Kirazli Y, Elmas F, Erdinç E. Do the benefits gained using a short-term pulmonary rehabilitation program remain in COPD patients after participation? Lung. 2007 Jul-Aug;185(4):221-5. doi: 10.1007/s00408-007-9011-4. Epub 2007 May 9. PMID: 17487535.
11. Ko FW, Dai DL, Ngai J, Tung A, Ng S, Lai K, Fong R, Lau H, Tam W, Hui DS. Effect of early pulmonary rehabilitation on health care utilization and health status in patients hospitalized with acute exacerbations of COPD. Respirology. 2011 May;16(4):617-24. doi: 10.1111/j.1440-1843.2010.01921.x. PMID: 21199163.
12. Lahham A, McDonald CF, Moore R, Cox NS, Rawlings S, Nichols A, Liacos A, Holland AE. The impact of home-based pulmonary rehabilitation on people with mild chronic obstructive pulmonary disease: A randomised controlled trial. Clin Respir J. 2020 Apr;14(4):335-344. doi: 10.1111/crj.13138. Epub 2020 Jan 11. PMID: 31880078.
13. Man WD, Polkey MI, Donaldson N, Gray BJ, Moxham J. Community pulmonary rehabilitation after hospitalisation for acute exacerbations of chronic obstructive pulmonary disease: randomised controlled study. BMJ. 2004 Nov 20;329(7476):1209. doi: 10.1136/bmj.38258.662720.3A. Epub 2004 Oct 25. PMID: 15504763; PMCID: PMC529363.
14. Nakamura Y, Tanaka K, Shigematsu R, Nakagaichi M, Inoue M, Homma T. Effects of aerobic training and recreational activities in patients with chronic obstructive pulmonary disease. Int J Rehabil Res. 2008 Dec;31(4):275-83. doi: 10.1097/MRR.0b013e3282fc0f81. PMID: 19008675.
15. Ringbaek TJ, Broendum E, Hemmingsen L, Lybeck K, Nielsen D, Andersen C, Lange P. Rehabilitation of patients with chronic obstructive pulmonary disease. Exercise twice a week is not sufficient! Respir Med. 2000 Feb;94(2):150-4. doi: 10.1053/rmed.1999.0704. PMID: 10714421.
16. Troosters T, Gosselink R, Decramer M. Short- and long-term effects of outpatient rehabilitation in patients with chronic obstructive pulmonary disease: a randomized trial. Am J Med. 2000 Aug 15;109(3):207-12. doi: 10.1016/s0002-9343(00)00472-1. PMID: 10974183.
17. Tsai LL, McNamara RJ, Moddel C, Alison JA, McKenzie DK, McKeough ZJ. Home-based telerehabilitation via real-time videoconferencing improves endurance exercise capacity in patients with COPD: The randomized controlled TeleR Study. Respirology. 2017 May;22(4):699-707. doi: 10.1111/resp.12966. Epub 2016 Dec 19. PMID: 27992099.
18. Vasilopoulou M, Papaioannou AI, Kaltsakas G, Louvaris Z, Chynkiamis N, Spetsioti S, Kortianou E, Genimata SA, Palamidas A, Kostikas K, Koulouris NG, Vogiatzis I. Home-based maintenance tele-rehabilitation reduces the risk for acute exacerbations of COPD, hospitalisations and emergency department visits. Eur Respir J. 2017 May 25;49(5):1602129. doi: 10.1183/13993003.02129-2016. PMID: 28546268.
19. Wadell K, Webb KA, Preston ME, Amornputtisathaporn N, Samis L, Patelli J, Guenette JA, O'Donnell DE. Impact of pulmonary rehabilitation on the major dimensions of dyspnea in COPD. COPD. 2013 Aug;10(4):425-35. doi: 10.3109/15412555.2012.758696. Epub 2013 Mar 28. PMID: 23537344.
20. Zambom-Ferraresi F, Cebollero P, Gorostiaga EM, Hernández M, Hueto J, Cascante J, Rezusta L, Val L, Anton MM. Effects of Combined Resistance and Endurance Training Versus Resistance Training Alone on Strength, Exercise Capacity, and Quality of Life in Patients With COPD. J Cardiopulm Rehabil Prev. 2015 Nov-Dec;35(6):446-53. doi: 10.1097/HCR.0000000000000132. PMID: 26252342.
